# Histone diversity in the archaeal domain of life

**DOI:** 10.1038/s41467-026-71849-3

**Published:** 2026-04-15

**Authors:** Shawn P. Laursen, Karolin Luger

**Affiliations:** 1 Department of Biochemistry, University of Colorado Boulder, Boulder, CO USA; 2 Howard Hughes Medical Institute, Chevy Chase, MD USA

**Keywords:** Histone variants, Nucleosomes, Archaeal genomics

## Abstract

Two-thirds of all archaea encode histones, proteins that are ubiquitously used to structure chromatin in eukaryotes. Archaeal histone sequences are less conserved than their eukaryotic counterparts, and insight into how they structure DNA is limited to a few species that fail to represent the archaeal domain. Here, we use bioinformatics, structure prediction, and molecular dynamics simulations to survey the diversity of histone-like sequences in archaeal genomes and to understand how they might interact with DNA. We identify five distinct types of histones that combine in seven strategies, involving either single histones, multiple histones of the same type, or combinations of types in each genome. Some strategies correlate with environmental pressures and some are phylogenetically restricted. Despite highly divergent sequences, structure predictions and simulations suggest similar histone-DNA binding modes for most strategies. Our work provides a guide to surverying diverse strategies for histone-based DNA organization in archaea.

## Abstract (web summary)

Archaea encode histone-like proteins that differ from eukaryotic sequences and among lineages. Here, the authors perform computational analyses to reveal five histone clusters in Archaea, which class into seven distinct strategies for DNA organization.

## Introduction

Histones are small, highly basic proteins consisting of three α helices connected by two short loops (the ‘histone fold’) that form either homo- or heterodimers via a ‘handshake motif’^1,2^. These proteins are present in genomes across all domains of life and are also found in some viruses^3–8^. The best-studied histones are from the eukaryotic domain, where heterodimers of histone H2B-H2A and H3-H4 assemble into an octamer that wraps 147 base pairs of DNA to form nucleosomes^3^. Eukaryotic histones are highly conserved and ubiquitously present across the entire domain. Homologs of the four types of histones are also encoded in the genomes of several ancient double-stranded DNA viruses that infect ameba^9,10^. The amino acid sequences of these histones are rather divergent amongst giant viruses, and differ in many ways from those of eukaryotes. While the overall topology of nucleosomes reconstituted from these viruses appears to be conserved (at least for the two distantly related viruses where this has been studied^6,7,11^), the individual histone chains can be found in a variety of tandem, triple, and even quadruple combinations and in truncated forms in different viruses^10^.

A subset of bacteria also has histone-like proteins, which were likely acquired through horizontal gene transfer^12^. While some of these are attached to other domains of mostly unknown function, many are standalone histones that are abundantly expressed and associated with the nucleoid^5^. Only two of these putative bacterial histones have been studied in detail, and their interaction with DNA is markedly different from that of eukaryotic histones. Histones from *Bdellovibrio bacteriovorus* create long protein-coated DNA filaments through ‘edge-on’ binding rather than wrapping the DNA to form discrete nucleosomes, although the binding mode on longer DNA is somewhat controversial^5,13,14^. A recent manuscript suggests yet another binding mode for a histone encoded by *Leptospira perolatii*^15^. Clearly, more research is needed to understand the role of histones in bacterial genome organization.

Histones are widespread in the domain of archaea. They were first discovered by John Reeve and coworkers in 1990^16^, and we now know that the majority of archaeal genomes encode at least one type of histone. As more archaeal genomes are discovered at a rapid rate through advances in sequencing and in wider sampling, there is ever more diversity to consider^17–19^. Archaeal histones exhibit much more sequence divergence than their eukaryotic counterparts^20^, which are amongst the most conserved proteins known^21^. Because archaea are found in many different and often punishing environments, their proteins must have evolved to cope with extreme conditions^22–26^. Unlike in bacteria, where histone genes are sparse, histones seem to be a deeply rooted feature of archaea, occurring in most higher taxa, with the notable exception of *Thermoplasmata* (formerly *Crenarchaeota*)^26–28^. A select few archaea encode histones with tails, with the potential for post-translational modifications. These organisms are mainly from the Asgard phylum, which is thought to be most closely related to eukaryotes^20,29^.

At least two closely related hyperthermophilic archaea, *Thermococcus kodakarensis* and *Methanothermus fervidus*, have histones that package DNA into so-called ‘hypernucleosomes’, best described as slinky-like assemblies. The geometry of the DNA superhelix closely mimics the superhelix formed by stacked eukaryotic nucleosomes, using near-identical features of the histones to engage the DNA backbone^4,30^. To date, research into archaeal histone-DNA complexes is limited to these two organisms (but see recently published manuscript^15^). In *T. kodakarensis*, histones contribute to transcription regulation^4^. Additional studies utilizing molecular modeling of sequences from methanogenic archaea and ChIP-seq in *Halobacterium salinarum* and *Haloferax volcanii* have begun to shed light on the function of histones in these organisms. A role for histones in global genome compaction has not yet been conclusively established, but these studies point toward a role in transcription regulation^4,31–34^.

Here, we parse the diversity of histone sequences in archaea by mining predicted protein databases. We group archaeal histones into five major clusters based on four biophysical properties (length, isoelectric point, hydrophobicity, and instability index). We then identify seven strategies for combining histones. Some genomes encode only a single histone, while other genomes harbor various combinations of histones. To understand possible co-dependencies between histones, we analyze the seven strategies separately, to allow us to tease apart, for example, whether basic histones that occur as the only histone in one genome have different features compared to those that co-exist with other basic or acidic histones. Finally, we predict the structure of the main histone combinations and inferred their ability to bind DNA using molecular dynamics simulations, providing a starting point for targeted structural and biophysical analysis.

## Results

### Archaeal histones can be grouped into five clusters

We first identified putative histones in the predicted proteomes of all 5869 available archaeal genomes in release 220 of the Genome Taxonomy Database (GTDB)^35^. Protein-coding sequences in this database were predicted from single genomic assemblies, likely representing unique species. Metadata, including sampling location, genome size, and GC content, were also calculated or collected. To identify histone sequences, we used HMMSearch with archaeal (PF00808) and eukaryotic (PF00125) histone PFAM models. We tested a variety of search strategies using different HMMer tools with a range of stringency cutoffs and found that HMMSearch with a liberal stringency captured most of the diversity found in the sequence space, without adding too much noise (Supplementary Fig. 1).

We then applied DBSCAN (Density-Based Spatial Clustering of Applications with Noise) to perform unsupervised clustering of the presumptive histone sequences, using the four easily calculated physical parameters with the most variance: length, instability index, isoelectric point (pI), and hydrophobicity/GRAVY score. Full clustering details can be found in the “Methods”. Briefly, we optimized the clustering parameters using small randomly sampled datasets, extracted the physical parameters that define each cluster, and used those bounds to label proteins in the overall dataset (Supplementary Table 1).

**Fig. 1:**
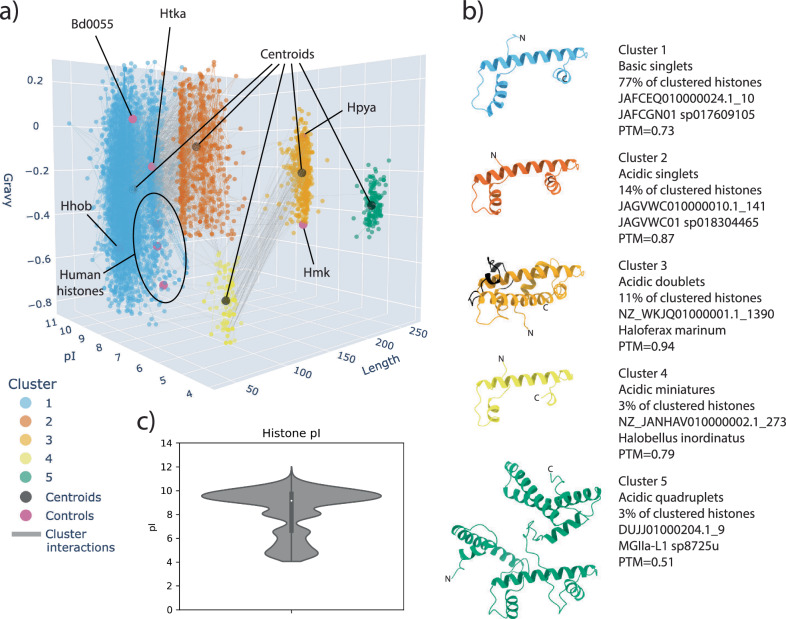
Archaeal histones can be grouped into five clusters. **a** Clustering of 6473 archaeal histone sequences using DBSCAN, plotted by length (number of residues), pI (isoelectric point), and GRAVY score (hydrophobicity). Sequences were clustered using these three dimensions plus a fourth, the instability index (not shown). Sequences closest to the center of each cluster in the four dimensions are denoted with a black dot; gene names for the centroids are listed in (**b**). Control histones (human histones H2A, H2B, H3, and H4, bacterial histone Bd0055, and archaeal histone HTkA) are indicated by purple dots. An interactive version of this figure can be found in the supplementary materials; all sequences are listed in the Source Data spreadsheets. **b** AlphaFold3 structure predictions of single chains of the centroids determined in (**a**), along with defining characteristic, gene name, genome, and AlphaFold3 PTM (confidence) score. N and C-termini are indicated. The linker region connecting the two histone fold domains in cluster 3 is shown in black. **c** Distribution of isoelectric points of all archaeal histones shown in **(a)**. A multimodal distribution can be observed around pI values of 10, 8, 6.5 and 4.5. 26.7% have an acidic isoelectric point. Isoelectric points around neutral are under-represented.

We used these physical parameters to group histones into five distinct clusters of histone-like proteins (Fig. 1a): basic singlets (cluster 1, blue), acidic singlets (cluster 2, red), acidic doublets (cluster 3, orange), acidic ‘miniatures’ (cluster 4, yellow), and acidic quadruplets (cluster 5, green). We selected the centroid sequence from each cluster and predicted their structures with AlphaFold3 (Fig. 1b, Table 1). Basic and acidic singlets form the characteristic histone fold that resembles the experimentally determined structure of the basic singlet HMfB, a histone from a well-characterized archaeon for which we have structural information (PDB 1A7W, 5T5K)^4,36^.

**Table 1:** Median values of physical parameters of histone clusters

Cluster	Type	# of Sequences	Length	pI	GRAVY	Instability	RMSD to 1A7W
1	Basic singlet	4969	70	9.5	−0.26	34	1.978
2	Acidic singlet	685	68	6.6	−0.11	34	1.055
3	Acidic doublet	536	143	4.7	−0.18	41	1.810
4	Acidic miniature	153	55	4.3	−0.60	43	3.310
5	Acidic quadruplet	130	262	5.2	−0.35	46	1.795

Number of sequences in each cluster, and median values for length of protein (amino acids), isoelectric point (pI), hydrophobicity (GRAVY score), instability index, and RMSD to histone HMfB from PDB 1A7W to the best aligning histone fold (Å) for each histone cluster were calculated after clustering in Fig. 1. See Supplementary Table 2 for ranges of values of each cluster.

Cluster 3 histones comprise two histone fold domains that are linked in a single polypeptide chain (colored in black in Fig. 1b), and they are predicted to form a structure that resembles the HMfB homodimer (PDB 1A7W). One representative of this cluster for which the structure is known is the acidic histone doublet from *Methanopyrus kandleri* (PDB 1F1E^37^).

The acidic miniature histone (cluster 4) is predicted to have an α2 helix that is shortened by one turn and also has a very rudimentary α3 helix, and in this it resembles the bacterial histone Bd0055 (PDB 8FVX, ref. ^5^), although the latter is positively charged overall. Finally, cluster 5 histones are unusual in that they consist of a long acidic chain with four predicted histone fold motifs. Archaeal histones have a bimodal distribution in terms of their charge: overall, a surprisingly large percentage (26.7%) of the 7157 predicted histones are acidic in character, while histones with neutral charge are largely absent (Fig. 1c). It is, however, possible that halophilic archaea are overrepresented in sequence space, and that the sequence distribution is currently skewed. As more and more archaeal genomes are published, the reported distributions are subject to change.

### Histones are present in seven different combinations in the archaeal domain

**Fig. 2:**
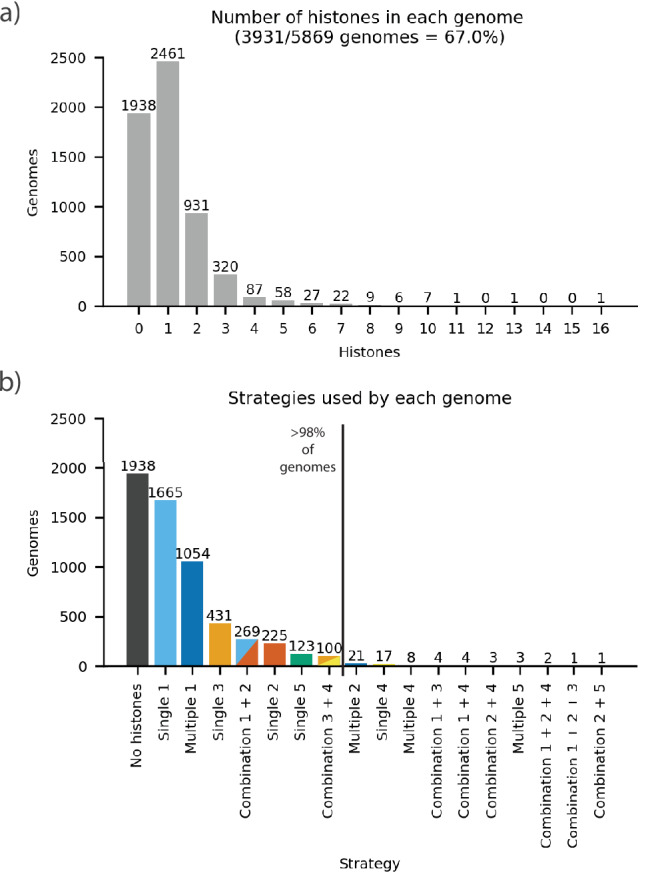
Histones are employed in seven major ‘histone strategies’ in archaea. **a** A distribution of predicted histone genes per genome. The majority of genomes contain three or fewer histones, and 33% of genomes encode no clearly identifiable histone (using our relatively conservative cutoff). **b** Distribution of genomes utilizing a particular histone strategy. These include genomes with only a single histone, genomes with multiples of the same type, or those with combinations of more than one type. Histone clusters are colored as in Fig. 1. This study focuses on strategies that are represented by 100 or more genomes (indicated by a vertical line).

According to our cutoff, of the 5869 archaeal genomes in the GTDB, 3931 (67%) encode at least one putative histone (Fig. 2a). Because each sequence represented in Fig. 1a is associated with a unique species, we were able to determine which genomes encode more than one histone and which combinations are the most prevalent. About 60% of all histone-encoding genomes have only one histone gene from either cluster 1, 2, 3, or 5 (Fig. 2a, b), and genomes encoding more than three histones are rare. We classify the genomes encoding only a single histone from a specific cluster as ‘single 1,2,3 or 5’, to separate them from genomes that contain different combinations of histones that also may include histones from the same clusters. Among genomes harboring more than one histone, genomes containing two or more histones from cluster 1 (basic singlets) are the most prevalent group, termed ‘multiple 1’ (the model organism *M. fervidus* is an example of this strategy). We also observe combinations of representatives from clusters 1 and 2, and clusters 3 and 4, termed combination 1 and 2 or combination 3 and 4, respectively (Fig. 2b, Supplementary Fig. 2). Representatives from cluster 4 (acidic miniatures) are almost always paired with an acidic doublet (cluster 3), and cluster 5 histones always occur as the sole histone-encoding gene. To simplify our analysis, we focused on general trends and restricted our further analysis to these seven most prevalent combinations of histones (single 1,2,3 and 5; multiple 1; combination 1 and 2; and combination 3 and 4), which represent *>*98% of histone-encoding archaeal genomes (indicated by a line in Fig. 2b).

### Some strategies are taxonomically restricted

**Fig. 3:**
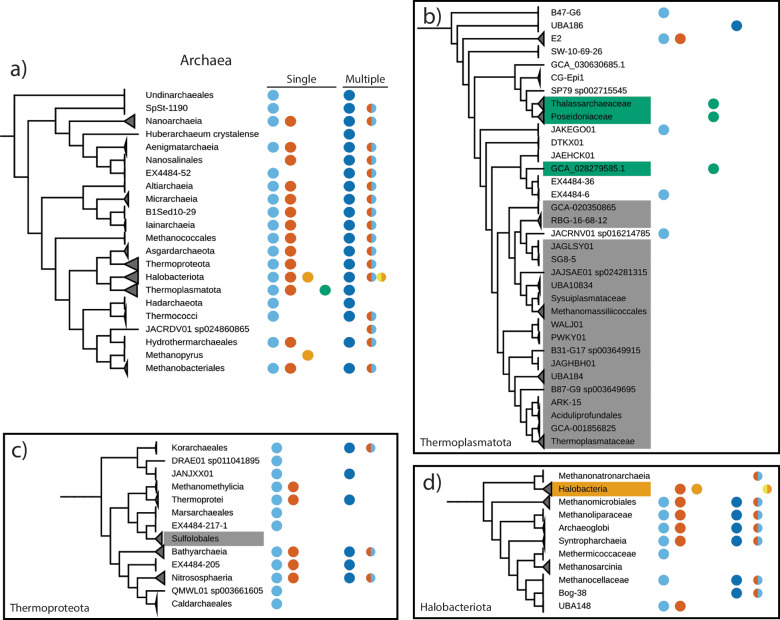
Some, but not all, strategies are taxonomically restricted. Colored dots indicate the presence of at least one genome in a taxon that employs a particular histone strategy. Histone clusters are colored as in Fig. 1. **a** Phylogeny of major archaeal groups. **b** Phylogeny of Thermoplasmatota, where most taxa do not encode histones (highlighted in gray). Genomes encoding histones from cluster 5 are exclusive to the order Poseidoniales (families *Thalassarchaeaceae* and *Poseidoniaceae*) and a single closely related genome (all highlighted in green). **c** Phylogeny of the Phylum Thermoproteota showing the absence of histones from the order Sulfolobales (highlighted in gray). **d** Phylogeny of Halobacteriota showing the exclusive presence of cluster 3 with cluster 4 (acidic doublet with acidic miniature histone) in the class Halobacteria (highlighted in orange), and the near exclusivity of the combination of single basic and acidic doublet histone, except for the genus Methanopyrus. More details are provided in Supplementary Fig. 4. An interactive and expandable tree containing histone annotations of the seven major strategies is available through iTOL: https://itol.embl.de/tree/1281386427164781716417921.

Histones that fall into cluster 1 (basic singlets) are widely dispersed across the entire domain of archaea and likely represent the typical archaeal histone, accounting for 77% of all clustered archaeal histones (Fig. 3a). They occur either as the sole histone or in combination with other basic singlets throughout the domain. These histones can be further divided into two clusters, which are distinguished by slightly different isoelectric points (median pI 9.6 vs 8 for Cluster 1-A and 1-B, respectively – Supplementary Fig. 13, Supplementary Table 1). Interestingly, no genome contains multiple histones from the 1-B sub-cluster, although they are found in combination with 1-A. Acidic singlets (cluster 2) are also pervasive, either as the only histone in the genome or paired with a basic singlet. In contrast, histones from clusters 3, 4, and 5 are phylogenetically restricted to specific taxa. In particular, histones from cluster 3 (acidic doublets) are mostly restricted to the class of Halobacteria, while representatives of cluster 5 (acidic quadruplets) are exclusive to members of the order Poseidonales (Fig. 3b).

Of the two most frequent combinations of histones, combination 1 and 2 (basic and acidic singlet) occurs in large groups in Methanobacteria and Halobacteria, and in smaller groups elsewhere in the domain (Fig. 3c, Supplementary Fig. 3). Combination 3 and 4 (acidic doublet and acidic miniature) is restricted to Halobacteria (Fig. 3d). We also confirmed previous findings that histones are exceedingly rare in the class Thermoplasmata (formerly Crenarchaeota) and in the order Sulfolobales (Fig. 3b, Supplementary Fig. 3)^23^. A full list of histones and their corresponding genomes can be found in the Source Data.

### Selective pressures may influence histone strategy

**Fig. 4:**
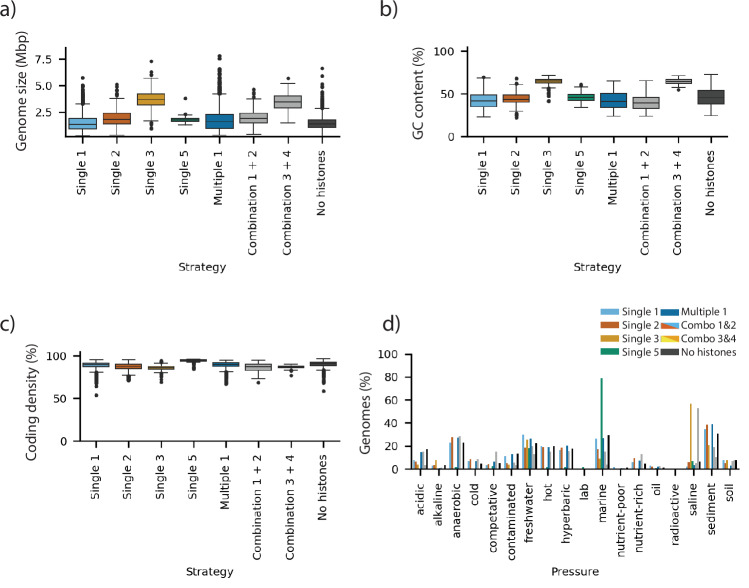
Correlation of histone presence and strategy with genome size, GC content, coding density, and environmental pressure. Differences in **a** genome sizes **b** GC content **c** gene coding density and **d** environmental pressures (Supplementary Table 2) of all archaeal genomes (*n* = 5879) in GTDB 220, broken down by assigned histone strategy. Boxplots display the median (center line), interquartile range (IQR; box bounds representing the 25th and 75th percentiles), and 1.5 × IQR whiskers (extending to the most extreme data points within 1.5 × the IQR from the box). Individual data points beyond the whiskers are shown as outliers (dots).

To understand the selective pressures associated with a specific histone cluster or strategy, we scoured metadata linked with the GTDB genomes for correlations. Specifically, we focused on genome size, GC content, coding density, and sampling location. Only two of the histone strategies (single 3 and combination 3 and 4) are found in genomes that are significantly larger and have a higher GC content than genomes that do not encode histones (Fig. 4a, b). This is probably because increased GC content is a known adaptation to high saline environments, and it is mostly halophiles that employ this strategy^38^. Protein coding density is somewhat higher in genomes encoding cluster 5 histones (single 5; Fig. 4c). Despite these subtle differences, our analysis does not explain why a subgroup of archaea does not appear to employ histones.

We also coded keywords found in genomic sample location annotations and found that some environmental pressures appear to correlate with specific combinations of histones (Supplementary Table 2). For example, archaea growing in anaerobic conditions tend to have combination 1 and 2 histones, and archaea found in extremely saline conditions seem to be enriched for combination 3 and 4 histones (Fig. 4d, Supplementary Fig. 4). It should be noted that these parameters are harder to quantify and verify, and as such, the correlations have to be taken with caution.

### Sequence bias and conservation of archaeal histones

To better understand the differences in physical parameters between all archaeal histones, we compiled the overall composition of amino acids in each histone cluster (Supplementary Fig. 5). There is an abundance of amino acids with a high propensity to form α-helices, such as alanine, isoleucine, leucine, and valine, as expected for histones, which are primarily α-helical. In the different histone clusters, we saw enrichment of, or bias away from, specific residues compared to the overall sequence composition of all archaea. Notably, archaeal histones outside of cluster 1 have acidic isoelectric points (Fig. 1c, Table 1). This is surprising as eukaryotic histones invariably have a positive overall charge and require basic residues (arginine and lysine) to effectively bind DNA in eukaryotes^39^. Besides an enrichment in acidic residues, the acidic histones from clusters 3, 4, and 5 exhibit the classic halophilic protein adaptation of a compositional bias from lysine to arginine^38,40^. Histones from these groups are also characterized by a higher percentage of the aromatic amino acids phenylalanine and tyrosine, which are both known to stabilize proteins in harsh environments (Supplementary Fig. 5d, e, h)^40^. Across all archaeal histones, tryptophan and cysteine are underrepresented compared to the universal proteome, perhaps due to their metabolically expensive nature^41^.

Overall, archaeal histones are much more divergent in their amino acid sequence than eukaryotic histones, which are among the most conserved proteins known^42^. The degree of conservation is particularly high in histones from halophilic organisms (cluster 3 and 4 histones), which could be due to a sampling bias towards closely related halophilic organisms, or due to a biological restriction to residues that facilitate function in hypersaline environments (acidic/aromatic residues, see above).

The cluster 1 and 2 histones that exist as the sole histone in the genome exhibit no characteristic sequence features compared to the histones that co-exist with others (Supplementary Fig. 3, compare panel a. b with e, f, and g). Similarly, cluster 3 histones have the same sequence features whether they occur alone or with cluster 4 histones. Cluster 4 is interesting as the ~150 members are almost universally conserved in length to 55 amino acids. These predicted proteins share many of the characteristic features with the bacterial histone Bd0055^5^ (Fig. 1b). Cluster 5 histones are unique in that they always occur as the sole histone-encoding gene, and their sequences are not well conserved. Aside from the first histone fold motif in these sequences, they do not contain many of the classic histone signatures (see below), suggesting that they may have co-opted the histone fold to perform a function in the cell not related to genome compaction or transcription regulation.

Specific ‘histone signature motifs’ are common to the majority of histones (boxed sequences in Supplementary Fig. 6, and shown for HMfB in panel j). The ‘RKTV motif’ is located in the L2 loop connecting helices α2 to α3. While the first three amino acids in this motif (RKT) are present in nearly all archaeal histones, V is often substituted by I or L, but is universally a hydrophobic amino acid. In all known histone structures from all domains of life, this loop pairs with the less conserved L1 loop of the second histone in the histone fold dimer to form the L1L2 DNA binding motif^2^. In the L1 loop, the ‘RV motif’ is found throughout the majority of archaeal histones. Valine packs against the conserved hydrophobic side chain in the L2 RKTV motif to stabilize the underside of the paired L1-L2 loop, and the arginine extends into the minor groove of DNA, which is stabilized by a threonine in the RKTV motif (the RT pair). We also note the strong conservation of a salt bridge that stabilizes the L2 loop in its critical conformation (the ‘R-D clamp’), which involves the arginine in the RKTV motif and a conserved aspartate, invariably located 7 amino acids downstream in the α3 helix of the histone fold, even in the rudimentary α3 helix in cluster 4 histones (Supplementary Fig. 6i). In eukaryotic, archaeal, and viral nucleosomes for which the structures are known, the fixed L1L2 configuration poises the main chain of both loops to contact the DNA phosphodiester backbone, and orients the arginine in the L1 loop (RV motif) to point into the compressed minor groove of the DNA. As such, these conserved amino acids in the L1-L2 loops represent a universal histone signature in addition to the ability to form histone fold dimers that might be useful to identify other histone-like proteins.

Unique to archaeal histones, a glycine in the L1 loop that we previously showed to be essential for hypernucleosome formation in *T. kodakarensis* histone HTkA^4^ is also highly conserved throughout histone clusters 1, 2, 3 (for class 3, only in the N-terminal histone domain), but not in clusters 4 and 5. This suggests that histone clusters 1–3 might be able to form closely stacked hypernucleosomes.

### Structural prediction of histone complexes: histone homo- and heterodimers

To predict whether multiples of histones might be used to bind DNA and fold into nucleosome-like structures, we used AlphaFold3 to build models of a representative histone from each strategy as dimers or as tetrameters^43^. To choose unbiased representative histone candidates for each of the seven strategies, we calculated the center of mass in the four dimensions used in Fig. 1 for each histone in each strategy from Fig. 2 and identified the sequence closest to that point. For genomes that encode two histones, we chose a genome that encodes the histone closest to the center of mass of one of the clusters and used both histones from that genome as representatives (Table 2).

**Table 2:** Representative genome for each strategy

Strategy	Representative Genome	Representative histone ID(s)	# of genomes
Single 1	*Nitrosotalea sp028867735*	JAGWFW010000004.1_41	1665
Single 2	*Methanococcoides*	JAIORJ010000016.1_63	225
Single 3	*sp021108185*	NZ_WKJQ01000001.1_1390	431
Single 5	*Haloferax marinum MGIIa-L1 sp8725u*	DUJJ01000204.1_9	123
Multiple 1	*JACPII01 sp016188175*	JACPII010000095.1_11, JACPII010000002.1_73	2782
Combination 1 and 2	*SZUA-1452 sp015662385*	Type 1 - DQUH01000053.1_3 Type 2 - DQUH01000042.1_12	269
Combination 3 and 4	*Halopenitus persicus*	Type 3 – NZ_FNPC01000002.1_292 Type 4 – NZ_FNPC01000016.1_27	100

Representative genomes were identified by encoding the closest histone to the average of all histones in each strategy, using the four physical parameters from Fig. 1. For strategies with multiple histones, genomes were chosen by calculating the closest to the average histones for each type within the strategy, and then choosing the genome that had the most prevalent composition of histones for that strategy (two histones for multiple 1, one of each for combinations 1 and 2 and 3 and 4). All amino acid sequences are found in Source Data.

The representatives from cluster 1, 2, and 3 form homodimers that resemble known structures of histone fold homodimers (Supplementary Fig. 7). The N and C termini of single 5 histones can adopt conformations similar to histone fold dimers through intra- and interchain interactions, respectively.

Basic histones that occur in genomes together with other histones are likely able to form both homo- or heterodimers (as shown experimentally for *M. fervidus*, an organism that employs the multiple 1 strategy)^44^. In the median organism representing this strategy, the second histone is missing a well-defined α3 helix, yet this histone is also able to homo- and heterodimerize in silico (Supplementary Fig. 7b). A number of histones from this cluster appear to have a prematurely terminated α3 helix, yet are still able to form homo- and heterodimers (not shown). In the median organism combining a basic and acidic singlet within its genome (combination 1 and 2), homo- and heterodimers are predicted with similarly high levels of confidence (Supplementary Fig. 7c). It is important to note that even the models of clusters with acidic charge maintain a basic putative DNA binding ridge on their outer surface. In our representative employing the combination 3 and 4 strategy, combining one acidic doublet with one acidic miniature, the doublet folds into a structure that is very similar to a canonical histone fold dimer. The acidic miniature histone from cluster 4 is predicted to fold into a homodimer that has a closer resemblance to the bacterial histone Bd0055 than to HMfA or HMfB.

### Some, but not all archaeal histone-fold dimers form tetramers via a four-helix bundle structure

The ability to form tetramers from histone fold dimers through well-defined four-helix bundle structures is a hallmark of all canonical nucleosomes^3,4,6^. This interface is formed by the pairing of the C-terminal end of the long α2 helix and the α3 helix of two separate histone dimers (Supplementary Fig. 8a, circled). We used AlphaFold3 to predict whether the histone fold dimers shown in Supplementary Fig. 7 are capable of forming tetramers through the four-helix bundle or any other means. Note that we display the solution with the highest level of confidence, with the acknowledgment that in some instances, alternative solutions are created with only slightly less favorable IPTM scores.

Representative histones from strategies using a single cluster 2 or 3 histone are all predicted to form homo-tetramers through canonical four-helix bundle assemblies that resemble archaeal HMfB and eukaryotic histones H2B-H4 and H3-H3’^3,4^. Basic singlets from ‘single 1’ may form closed tetramers, as well as open, canonical tetramers. Closed tetramers are oligomeric histone assemblies in which one histone dimer contacts another dimer along the entire face of the dimer instead of just contacting through one four-helix bundle (compare, for example, “Single 1” to “Single 2” in Supplementary Fig. 8b). This is the case for our median histone sequence from the single 1 genome. Note that such closed tetramers have not been observed experimentally for any archaeal histone with the typical 28 amino acid long α helix, while open tetramers have been visualized in various complexes with DNA^4,30,45^. In our experience, these predictions have to be taken with a healthy dose of skepticism: for example, for HMfB, for which structures are known, AlphaFold3 predicts a closed and open tetramer as well as a ‘back-to-back tetramer’ that doesn’t involve the four-helix bundle with closely spaced confidences . Nevertheless, it is predicted to generate an open tetramer resembling the experimentally determined structure when in the presence of DNA (see below). No combination of histone fold dimers from the single 5 representative is predicted to form higher-order assemblies mediated by a four-helix bundle (Supplementary Fig. 8b, green).

Our representative for a multiple 1 genome encodes two basic histones, here referred to as “histone A” and “histone B”. Only histone A is predicted to fold into the canonical tetramer. Histone B alone or combined with histone A, does not form a canonical tetramer in silico, but we did not explore whether this is a general feature of all histones in this cluster (Supplementary Fig. 8c). Histones from combination 1 and 2 (basic and an acidic singlet; a wide-spread combination) are predicted to form open tetramers either from the basic histone alone, or from basic-acidic histone fold dimers (Supplementary Fig. 8d). The acidic histone fold homodimer can form a tetramer through a variety of arrangements with nearly the same confidence (inset). Finally, the combination of an acidic doublet and an acidic miniature (combination 3 and 4), specific to and prevalent in halophiles, is not predicted to form a heterotetramer. While the acidic doublet forms an open ‘tetramer’ structure, the acidic miniature assembles into either ‘back-to-back’ (shown) or face-to-face tetramers with similar confidence.

### AlphaFold3 predictions and simulations suggests that most histones form stable structures with DNA

To explore whether these systems might form plausible complexes with DNA, and in particular whether they might be capable of hypernucleosome formation, we employed AlphaFold3 to predict structures with DNA and then evaluated their physical stability using all-atom molecular dynamics simulations. We predicted nucleosome models with the equivalent of 8 histone folds from each histone strategy and 147 bp DNA, sufficient for forming a nucleosome-like arrangement. We chose to run these predictions (and the simulations described below) with the canonical 147 base pair DNA fragment derived from Widom 601 for the following reasons: first, we wanted to provide enough length to probe the ability of archaeal histones to compact DNA in a closed hypernucleosome^4^. Second, the Widom 601 sequence has a strong propensity to form nucleosomes in vitro. Third, because the sequence is the most prevalent in deposited nucleosome structures, models generated by AlphaFold3 using it as input are biased toward nucleosome formation. As such, using this sequence would allow us to identify histones that are emphatically unable to form these compacted structures due to steric or electrostatic repulsion between two ‘layers’ of the hypernucleosome, and would allow for a direct comparison to eukaryotic nucleosomes.

All combinations that are able to form canonical, open tetramers via the four-helix bundle are predicted to wrap DNA around the outside of the histone torus (Supplementary Fig. 9). The only cluster 3 acidic doublet histone tetramer with known protein structure (*Methanopyrus kandleri* histone NC_003551.1_1799) is predicted to behave like the representative single 3 histone (shown in Supplementary Fig. 9b) in that it is predicted to form mostly open, destabilized structures (Supplementary Fig. 12). Cluster 4 and 5 histones are not predicted to wrap DNA. Note that the IPTM scores are rather low for all models except for those with combinations 1 and 2 (Supplementary Fig. 9d).

**Fig. 5:**
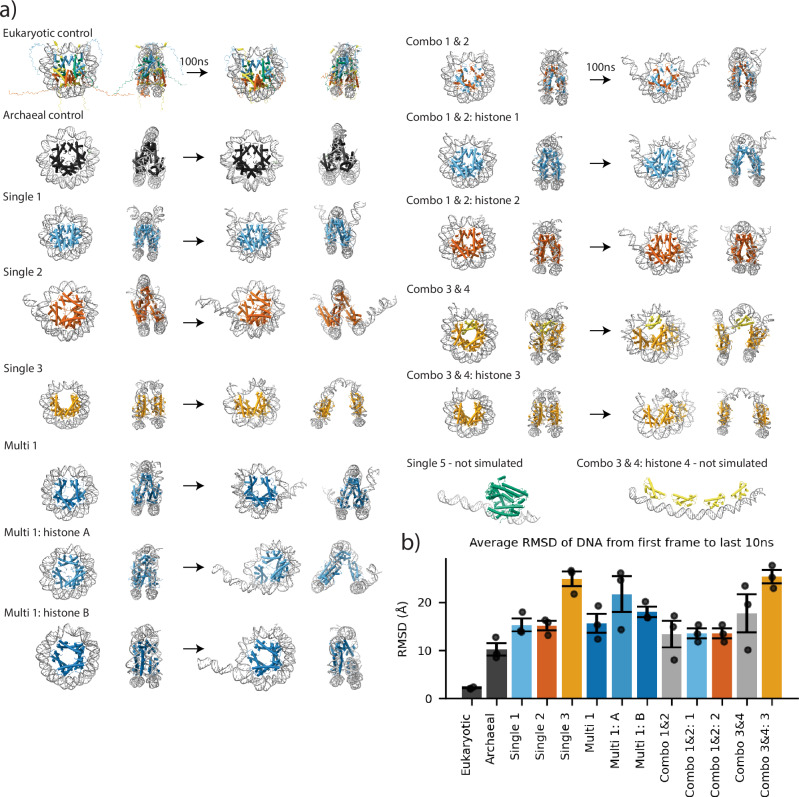
Molecular dynamics simulations suggest that not all of the predicted nucleosome-like structures are stable. **a** All-atom molecular dynamics simulations of nucleosome-like particles predicted from each histone strategy (median representative, Table 2), in isolation or in combination. Simulations were started from an AlphaFold3 prediction shown in Supplementary Fig. 9, using the equivalent of eight histone folds and 147 bp of Widom 601 double-stranded DNA. AlphaFold models of the eukaryotic nucleosome and a nucleosome constructed from HMfA were used as controls. Simulations were run for 100 ns in triplicate. Side and face views of the starting model and the representative final model are shown. **b** Change in DNA topology from the beginning to the end of the simulation. RMSDs (Å) were calculated by averaging the RMSD of the DNA in each structure over the last 10 ns of the simulation to the starting frame. Because the DNA represents the topology of a nucleosome and is common to all the structures, we reasoned it was the most consistent way to monitor how much the models changed over time. Error bars represent the standard error of the mean over three replicate simulations.

We ran molecular dynamics simulations of all structures where nucleosome-like structures were predicted for 100 ns in triplicate, to allow for relaxation and sampling of conformational flexibility. Our goal was to determine whether the AlphaFold3 models shown in Supplementary Fig. 9 are energetically plausible. In these simulations, the human nucleosome and the archaeal structure from HMfB (for which there is a structure on shorter DNA, PDB 5T5K) remained tightly wound and experienced little conformational change, as judged by minimal movement of DNA during the simulation (Fig. 5b). The median representative histone from an organism employing the same strategy (multiple 1) also formed stable nucleosome-like structures. Acidic single 2 histones form plausible structures, although they seem somewhat destabilized compared to the structures formed by the basic histones. Structures predicted with the single 3 histone cluster unraveled, eventually losing the protein-protein interactions crucial to maintaining a tightly wound nucleosome-like structure. As the original median histone formed an open structure, we also simulated a second nucleosome of this type, which was predicted to form a closed structure (shown in Supplementary Fig. 9), but both simulations resulted in similar ‘final’ structures with open conformations. Nevertheless, even these acidic cluster 2 and cluster 3 histones maintain their interaction with DNA throughout the simulation. Remember that single 2 and single 3 strategies are mostly found in halophilic organisms. Indeed, when we repeated simulations for these complexes in 2 M KCl, these structures remained closed (Supplementary Fig. 10).

We also ran simulations of nucleosomes from genomes that encode multiple histones. We analyzed these histones in isolation and in combination (Fig. 5), allowing us to query whether they might require a partner to form stable nucleosomes. For the two basic histones from the genome used to represent multiple 1 genomes, nucleosomes made from the combination of both histones or just histone B remained closed over the simulation. In contrast, nucleosomes made from just histone A appeared unstable (Fig. 5a, b). This could suggest a means by which accessibility to the genome might be regulated, an idea that was recently supported by experimental data. When repeated in 2 M KCl, these histone A nucleosome structures remain in the closed state throughout the simulation, maybe also hinting at environmental regulation of nucleosome state (Supplementary Fig. 10).

As predictions were unable to assemble both histones from combination 3 and 4 into a nucleosome structure, we manually placed the cluster 4 histone (which co-occurs with cluster 3 histones) into the obvious space made when predicting the nucleosome with only cluster 3 histone dimers. We also simulated a nucleosome from cluster 3 histones in isolation. In the end, both simulation strategies resulted in unstable structures, except in one of the three combination 3 and 4 simulations, where a wrapped conformation was maintained throughout the duration of the simulation. Whether this acidic doublet combines with the acidic miniature histone to form a nucleosome-like structure or not remains to be experimentally tested. Likely, these structures require precisely oriented histones or high ionic strength to function properly, as organisms encoding them often utilize a “salt-in” strategy to cope with high levels of extracellular salt.

The prediction of just the cluster 4 or cluster 5 histones alone did not result in nucleosome-like structures. If anything, these investigations highlight the limitations of AlphaFold3 in the prediction of histone assemblies beyond histone fold dimers and emphasize the requirement for at least some degree of energy minimization.

## Discussion

Archaea are a diverse group of organisms that have adapted to a wide and extreme range of environments. With billions of years of evolution, this domain of life has diversified to meet unique challenges, and these adaptations presumably include strategies to protect and package genomes. This is particularly relevant for organisms that thrive at extreme temperatures, pH, and ionic strengths, all presenting challenges to genome integrity. While some archaea package their genomes exclusively with non-histone proteins such as Cren7, Alba, or Sul7d, the majority of them rely on histones^28^, although the extent of their involvement in genome organization is not clear at this point. Our search of available archaeal genomes reveals that 67% of known archaea encode putative histones that we group into five clusters depending on length, charge, hydrophobicity, and instability index. Because we used a rather conservative cutoff, it is likely that this percentage could be higher. The majority of histones are predicted to comprise a single, mostly tail-less histone fold domain with basic charge.

Four out of five clusters of archaeal histones (26.7% of all histones) are acidic in character; they are either of canonical length (cluster 2), encode multiple histone folds in a single chain (cluster 3 and 5), or are predicted to have shorter α2 and α3 helices, resembling bacterial histones at least architecturally, if not in overall charge (cluster 4). Histones are encoded in genomes either by themselves or along with other histones, most commonly with one other histone of the same cluster, or combining two histones from different clusters. Intriguingly, 269 genomes encode an acidic and a basic histone that have <60% sequence identity, displaying a diversification in histone sequences similar to what might have happened during the diversification into H2A, H2B, H3 and H4 from a single histone precursor in early eukaryotes^12^.

Using the structure prediction tool AlphaFold3, we predicted the structures of the ‘median histone’ for each cluster/strategy, in different oligomerization states and in complex with DNA. We deliberately chose the median histones rather than the nearest model organism to avoid bias and to best represent each cluster and strategy. Many of these histones are derived from metagenomes, and the corresponding organisms have not yet been cultivated.

Our analyses suggest that four out of the five clusters form canonical histone fold dimers, most of which tetramerize via a four-helix bundle interface that is a hallmark of eukaryotic histone interactions. Representatives of clusters, either alone or in combinations, that are predicted to tetramerize via a four-helix bundle are also predicted to organize DNA into nucleosome-like structures that remain stable in molecular dynamics simulations. These structures are similar to the experimentally determined structure of a cluster 1 histone in complex with DNA, which we showed forms a ‘hypernucleosome’ that may flex and open stochastically^4,30^. Histone-DNA interactions are maintained throughout the simulations for representatives of most clusters and strategies, even for those that have an overall acidic character. This is probably because even these highly acidic proteins maintain the ‘basic ridge’ around their outside that serves as a DNA-binding surface. Our simulations have not yet considered the diverse environments that our ‘median organisms’ might dwell in. For example, cluster 3 histones are mostly found in halophiles, and as such, the simulations at high (> 2M) ionic strength may be a better predictor of their histone-DNA complexes. The requirement for high ionic strength for halophilic proteins to interact with DNA was demonstrated with the transcription factor RosR, in which molar quantities of monovalent salts were necessary for tight interaction and crystallization with DNA^46^. Our data suggest that cluster 5 histones, even though they form plausible histone fold dimers, might not bind DNA, and the role of cluster 4 histones (shorter acidic histones of highly restricted length to 55 amino acids, co-occurring with cluster 3 histones) remains unresolved. Importantly, given the limitations of AlphaFold (also demonstrated here), all predictions have to be verified experimentally.

While eukaryotes encode a narrow and conserved set of four histone sequences (plus a variety of histone variants^8^), putative archaeal histones exhibit much higher sequence diversity and combine them in a number of ways. Nevertheless, the vast sequence space has brought into focus universal, functionally linked histone signatures, the RKTV motif and the R-D clamp in the L2 loop of the histone fold, the RV motif in the L1 loop, and the RT pair (Supplementary Fig. 8j). In combination, these motifs serve to rigidify the L1L2 pairing to allow it to make main-chain interaction with the phosphodiester backbone of the DNA, and to orient an arginine to protrude into the compressed minor groove of DNA (referred to as a sprocket arginine)^47^. These signatures have been described over 25 years ago, and are reinforced here in a vastly expanded sequence space. The diversification in histone sequence outside of these motifs likely allows archaea to adapt to a more diverse and extreme set of intracellular conditions than could not be tolerated by eukaryotic systems, and might afford them the ability to live in these environments without compartmentalizing their genomes. It must be emphasized, however, that a role for archaeal histones in genome compaction has not been established even in the few model organisms that have been studied to date. Rather, it appears that in many cases histones might function as transcription factors instead^4,31,33,48^. Answering this question requires the adoption of techniques commonly used to study genome organization in eukaryotes to the wide variety of archaeal organisms, many of which cannot even be cultured at this stage.

Recently, other groups have used different tools to sample histone diversity across both archaea and bacteria. In a study by Dame and colleagues, histone sequences from archaea and bacteria were clustered into different groups based on sequence features^14^. This approach led the team to focus mainly on an array of bacterial histone sequences that are fused to other functional domains and whose functions are largely unknown. The work highlighted the power of approaches like HMMSearch to find disparate sequences that may fold into similar structures. That study utilized a different sequence database than ours, so we attempted to use their methods to compare whether our DBSCAN method would find similar or disparate clusters. We found that clustering by sequence using the CLANS method did not produce the same clusters as our method (Supplementary Fig. 11). We compared our overall findings to those of the other study in Supplementary Tables 3, 4. It should also be noted that the current archaeal sequence space might be skewed towards extremophiles, and there is much to be discovered in terms of archaeal histone sequence diversity.

Our study emphasizes the need to explore these understudied and diversified classes of proteins and to explore the biology of organisms that may otherwise be overlooked. By selecting organisms that broadly sample the diversity of archaeal histones, we can allocate resources strategically to maximize discovery. As many of the organisms have never been cultured, a logical next step to this work is to use structural biology and biochemistry to uncover how these histones physically structure DNA. An intriguing addition to the sparse availability of experimental structures has recently been published as a preprint, and suggests subtleties of archaeal chromatin structures that are caused by variations in histone sequence. As recent breakthroughs in culturing (and, one would hope, genetically manipulating) archaea are revolutionizing the field^49–52^, hypotheses gained from biophysical characterization could eventually be put to the test in the cell.

Our work highlights the power of structural prediction tools such as AlphaFold, yet demonstrates that these cannot (yet) replace experimental structures and biophysical analyses. To use these predictive tools properly, context and prior knowledge are necessary to avoid over-interpretation. For example, AlphaFold predicted the tetrameric structures of many histones to adopt conformations that appeared ‘closed’, yet when reinforced with a DNA sequence that is biased in the PDB to form nucleosomes, these same histones formed nucleosome-like structures. AlphaFold and similar tools are built on massive amounts of training data and usually do well when re-predicting structures they have trained on. Some models ignore the basic laws of physics, placing atoms on top of other atoms and predicting structures that fall apart in molecular dynamics simulations (Fig. 5). At least for now, and for this system, the predictions are not yet ready to stand on their own without experimental validation, especially for the more complex models beyond histone tetramer, and in the presence of DNA.

## Methods

### Histone identification and HMMSearch optimization

Predicted archaeal protein sequences were downloaded from GTDB, release 220 (https://gtdb.ecogenomic.org/). This dataset included 11,277,496 proteins from 5869 genomes, each with a specific taxonomic lineage. 7140 putative histones were identified using an HMMsearch against PF00125 (PFAM for eukaryotic histones) and PF0808 (PFAM for archaeal histones). To establish which confidence thresholds to use with the JackHMMER and HMM- Search, we screened a range of expectancy values (E-Value) for each search strategy that went low enough to collect no hits and went high enough to be limited by filters built into the HMM algorithm (Supplementary Fig. 1b). We noticed that most of the search strategies slowly collected hits up to an inflection point, where the number of hits began to increase rapidly. We reasoned that after this point, the search models return mostly noise sequences. By iteratively clustering around this inflection point, we were able to determine that hits above these E-values mostly constituted noise. Although most hits at the inflection point overlapped between search strategies, small outlier groups existed, so we combined the hits from both PFAMs around the inflection point and performed the rest of our analysis on this set (Supplementary Fig. 1c). We eventually used E = 4.0 for PF00125 and E = 0.1 for PF00808.

### DBSCAN clustering

Histone sequences were imported with associated metadata from the GTDB. Ambiguous sequences were filtered out. Physical parameters of sequences were calculated using ProtParam from the Bio.SeqUtils Python package. Histones were clustered with DBSCAN (implemented through the SciKitLearn package), optimized for a silhouette score of 0.25 (*e* = 0.5 *n* = 40) on the four parameters with the highest variance: length, pI, GRAVY, and helical propensity. Parameters were standardized prior to clustering using z-score normalization. To determine these clustering parameters, the data were randomly sub-sampled and tested with a range of parameters to optimize the silhouette score (Supplementary Fig. 1). 0.25 was chosen as a target silhouette score, as it was able to reproduce clusters reliably after many rounds of clustering. After optimizing, the parameters were applied to two additional data subsets, verifying that the same number of clusters of roughly the same size were found in each. The physical parameters of each cluster were then calculated from the three subsets to define boundaries for the whole dataset. These ranges were tested on another three random subsets that were independently clustered to verify that the labels matched 95% of points in each test. The verified ranges were then used to label all points in the overall dataset. Proteins that failed to cluster into one of the five groups were removed from further analysis. Centers of mass and nearest neighbors were calculated for each cluster in standardized space and mapped back to real space. Edges were mapped, linking histones coming from the same organism. Histones were then sorted into genomes, and common strategies were calculated. Taxonomic data from GTDB were then used to map histone strategies onto a taxonomic tree using iTOL^53^.

### Metadata correlation

After assigning histones to strategies, a practical cutoff of 100 histones per group was applied to simplify analysis. Histones from groups that did not meet the cutoff were not used for further analysis, but were still included in the database. Metadata associated with each strategy were aggregated and comparisons to genomes without histones (No histones group) were preformed using the Shiparo test from the SciPy.stats Python package. We chose this test to deal with comparisons between datasets containing uneven variances. We chose metadata that we felt were most relevant to understanding the presence of histone: genome size, genomic GC percentage, and gene coding density.

### Environmental pressure correlation

We manually extracted the location data associated with each genome and coded keywords in each location to a set of standardized locations, which encompassed most of the genomes in the dataset. We then associated each of these locations with the environmental pressure(s) they most likely impart. A full list of keywords and coding can be found within the scripts.

### Sequence conservation

Conservation of histones from each group was calculated by taking the average occupancy at each position of aligned histones (aligned with MUSCLE) using a custom Python script^54^. ‘Highly conserved’ residues represent residues whose conservation is at least one standard deviation greater than the mean conservation for that alignment. Conservations were calculated for each type of histone, both before and after strategies were assigned.

### Compositional bias

Amino acid composition of histone groups was calculated in Python using NumPy and plotted using Matplotlib. Composition was calculated on a per-residue basis, not as an aggregation of all the residues from all histones in a group. Because most histones in each group were of similar length, this normalization did not have a drastic effect, but still seemed appropriate to correct for a bias towards the composition longer sequences.

### Structural prediction

We predicted the structures of histones from each strategy as dimers (two histone folds), tetramers (four histone folds), and nucleosomes (with the addition of 147 bp of dsDNA) using AlphaFold3, as implemented through the online server. We visually inspected each of the five models outputted by AlphaFold3 and proceeded with analysis on the highest confidence model not containing major clashes (usually the highest confidence model, i.e model 0). IPTM scores are reported in the figures.

### Molecular dynamics simulations

AlphaFold3 nucleosome predictions were used as starting models for simulations. Models were prepped for simulation using ChimeraX^55^. The terminal phosphate from each DNA strand was removed (to prevent simulation errors later), models were protonated, and then subjected to a few frames of MD implemented by using the ”Tug” function in ChimeraX and pulling on a single hydrogen atom at the terminus of a DNA strand. This ”Tug” step allowed the AlphaFold3 model to relax atoms and resolve clashes orders of magnitude faster than doing the same by hand. No gross topological changes were observed. All-atom molecular dynamics simulations with explicit solvent were carried out using AMBER and the ff14SB, bsc1, and tip3p forcefields (for protein, DNA, and water, respectively)^56^. Structures were protonated again through TLEAP and hydrogen mass repartitioned in PARMED. Structures were placed in cubic boxes surrounding the structures by at least 25 ˚A, charge neutralized using potassium and chloride ions, potassium ions, and hydrated with water molecules. The structures were energy minimized in two, 5000 step cycles: the first restraining the protein and DNA molecules to allow solvent relaxation and the second to allow full system relaxation. Minimized structures were then heated to 300 K and slowly brought to atmospheric pressure (1.01325 atm). The systems were then simulated for 100 ns in 4 fs steps. Simulation were performed in triplicate by starting the simulation over using a different random number during the heating phase. Distances between phosphates on neighboring residues at the center of a DNA strand were calculated as a proxy for nucleosome unfolding in representative simulations.

### Reporting summary

Further information on research design is available in the Nature Portfolio Reporting Summary linked to this article.

## Supplementary information

Supplementary Figs.

Reporting Summary

Transparent Peer Review file

## Source data

Source data

## Data Availability

Source data are provided with this paper. All underlying protein sequences and metadata were collected from GTDB, specifically release 220. Histone sequences and classifications are provided in Source Data (Excel spreadsheets). Accession codes used in this work: PF00808, PF00125, PDB 1A7W; PDB 5T5K; PDB 1F1E; PDB 8FVX. Source data are provided with this paper.

## Code availability

[https://github.com/shla9937/archaeal_histone_diversity] and Zenodo [10.5281/zenodo.18932309].
