# Supplementary material for: Histone diversity in the archaeal domain of life
Source: Nat Commun. 2026 Apr 15;17:5254. doi: 10.1038/s41467-026-71849-3 (PMC13260811; doi:10.1038/s41467-026-71849-3)
Supplement: Supplementary file 1 — Supplementary Figs. [file 41467_2026_71849_MOESM1_ESM.pdf]

Histone diversity in the archaeal domain of life

Supplementary Material

Supplementary Table 1. Ranges collected from DBSCAN clustering used to label histones. Ranges of physical parameters used to define each histones cluster in Figure 1. Physical parameters used are protein length (residues), isoelectric point (pI), hydrophobicity (GRAVY), and instability index.

Cluster	Length	pI	GRAVY	Instability index
1	37.5 - 113.5	7.7 - 11.6	-0.83 - 0.27	4.5 - 68.9
1-A	61 - 84	8.6 - 11.1	-0.78 - 0.25	6.2 - 63.0
1-B	62 - 78	7.7 -8.4	-0.53 - 0.14	8.0 - 59.6
2	46 - 95.5	4.5 - 6.9	-0.43 - 0.21	16.5 - 53.6
3	134.5 - 165	4.3 - 6.1	-0.43 - 0.05	27.9 - 54.8
4	46 - 73.5	4.1 - 5.4	-0.78 - (-0.45)	29.9 - 55.7
5	258 - 277	4.8 - 6.0	-0.52 - (-0.15)	35.2 - 58.7

Supplementary Table 2. Keywords used to parse genomic sampling locations from GTDB data.

Keywords in location data	Location label	Associated pressure(s)
acid, ph 4, ph 3	acid	acidic
soda, alkaline	alkaline	alkaline
seep	cold seep	hyperbaric, cold, anaerobic
permafrost	permafrost	cold
salt, brine, saline	salt	saline
hot spring, hot pool, volcano, hot solfataric spring, geothermal	hot spring	hot
mine, mining	mine	contaminated, acidic
gut, intestine, rumen, ruminant, caecal, sheep, intestinal, caecum	gut	acidic, competitive
vent, hydrothermal, chimney, black smoker	hydrothermal vent	hyperbaric, hot, anaerobic
nuclear, olkiluoto	nuclear	radioactive, contaminated
sediment, mud	sediment	sediment
compost	compost	nutrient-rich
anaerobic digester, bioreactor, digestion, biodigester, reactor, fermentation	bioreactor/digester	nutrient-rich, anaerobic
waste, wwtp, sludge	waste	nutrient-rich, anaerobic, contaminated
landfill	landfill	nutrient-rich, anaerobic, contaminated
feces, fecal, stool, faeces, manure	feces	nutrient-rich, anaerobic
mouth, oral, dental	oral	competitive
estuary	estuary	marine, freshwater
ocean, marine, sea, atlantic, intertidal, tide-pools, bay, deep, depth, coastal, pacific	marine	marine
soil, grass	soil	soil

groundwater, well, aquifer, spring water	groundwater	freshwater
(not marine) + water, aquatic, wetland, river, pond, peatland, lake	water	freshwater
biofilm	biofilm	competitive
oil, petro (but not with soil)	oil	oil
lab	lab	lab
frac	fracking	oil
rock, aspo hrl	rock	nutrient-poor
none, metagenome, ""	none	none
(fallback case)	other	other

Supplementary Table 3. **Comparison of this study to the analysis performed in Schwab et al.** (reference 14). The latter study used different methods to probe a different database. This table outlines the key similarities and differences.

	This study	Schwab et al., 2024
Domains of life	Archaeal	Archaeal and Bacterial
Database	GTDB 220 (11M proteins)	InterPro (5M proteins)
Clustering method	DBSCAN	CLANS
Clustering properties	Physical parameters (Length, pI, hydrophobicity, instability index)	Sequence
Number of histones found	7,157 (archaeal)	~4400 (archaeal), ~1500 (bacterial)
Number of clusters found	5 clusters (7 combinations)	17 clusters (from Archaea and Bacteria)
Analysis performed	Histone identification, clustering, co-occurrence in genomes, taxonomic prevalence, association with environmental pressure, amino acid compositional bias, residue conservation, structural prediction, molecular dynamics simulation	Histone identification, clustering, structural prediction (protein only), gene clustering, phylogenetics, crystallography
Major conclusions	Archaeal histones are clustered in 5 putative histone families. Members co-occur in 7 major combinations.<br>Some likely form hyper-nucleosome structures<br>Provides a means to target histone diversity in archaea.	Archaea and bacteria encode 17 difference histone types, many types have co-opted other protein folds in bacteria

Supplementary Table 4. **Comparison of histone clusters found in this study to those identified in Schwab et al. 2024 (reference 14).** Histone clusters that represent similar proteins are juxtaposed. Because different protein databases were used, direct comparison of cluster overlap is not possible. The two clusters with good agreement are Cluster 3 & Halo double; and Cluster 5 & Poseidoniia double.

Laursen 2025	Schwab 2024
Cluster 1 (most do not include tails)	Nucleosomal (most include tails)
Cluster 2	Methanococcales (~20 residues longer)
Cluster 3	Halo double
Cluster 4 (exclusive to halophiles)	Face-to-Face (not exclusive to halophiles)
Cluster 5	Poseidonia double

Supplementary Figure 1

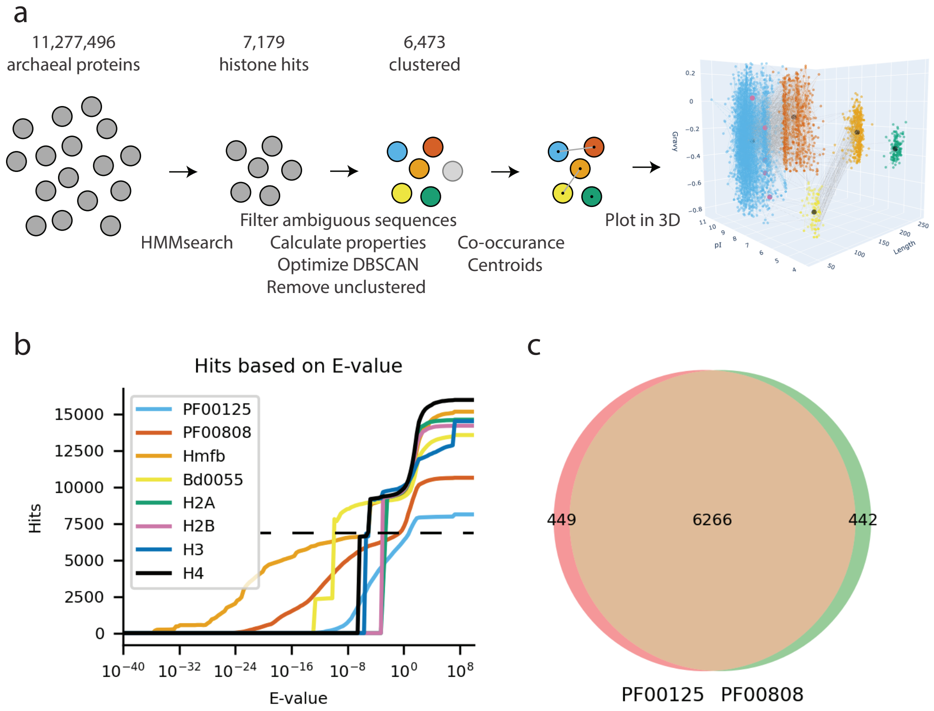

Supplementary Figure 1: Iterative clustering strategy to generate Figure 1. a) All archaeal protein sequences from the GTDB (v220) were collected and selected for homology to archaeal and eukaryotic histones using HMMsearch against various inputs (outlined in b). Matching sequences were then filtered, physical protein parameters calculated, clustered using DBSCAN, and then unassigned sequences were removed. Co-occurrence networks (proteins in the same genome) were defined and centroids for each cluster were calculated based on nearest neighbor to center of mass of each cluster. The resulting data were plotted against three of the four features used to cluster them and colored according to cluster (1 – blue, 2 – red, 3 – orange, 4 – yellow, 5 – green). **b)** Number of hits obtained for a given E-value for HMMsearch (PF00125 and PF00808) or JackHMMer (Hmfb, Bd0055, H2A, H2B, H3 and H4 against all archaeal protein sequences. The inflection point denoted by the dashed line gave reproducible clusters when clustered over three randomly sampled subsets and was used to define histone hits for analysis. **c)** Overlap between histone sequences returned by HMMsearch using PF00125 (eukaryotic histones) and PF00808 (archaeal histones). Although there is a high degree of overlap, ~440 were unique to either archaeal or eukaryotic histone searches. Combining these two datasets resulted in robust clustering and captured the majority of diversity observed in the other search strategies.

Supplementary Figure 2

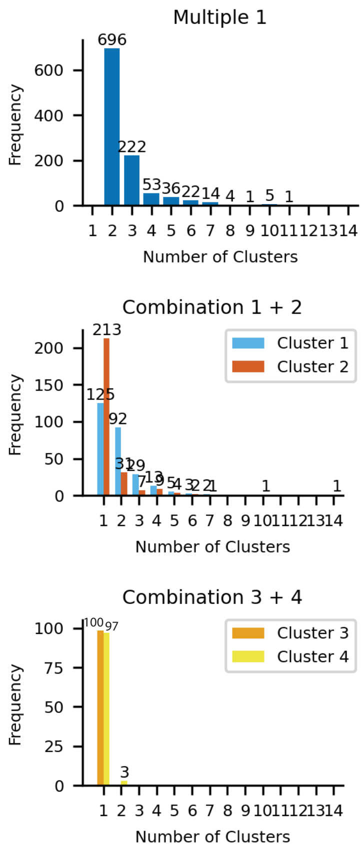

Supplementary Figure 2: Breakdown of the number of each type of histone within genomes encoding multiple histones. Most genomes with multiple histones encode two. Combination 1&2 genomes often contain unequal ratios of cluster 1 to cluster 2 histones. Genomes from combination 3&4 encode cluster 3 histones at a 1:1 ratio with cluster 4 histones, except for three cases where only two cluster 4 histones are present.

Supplementary Figure 3

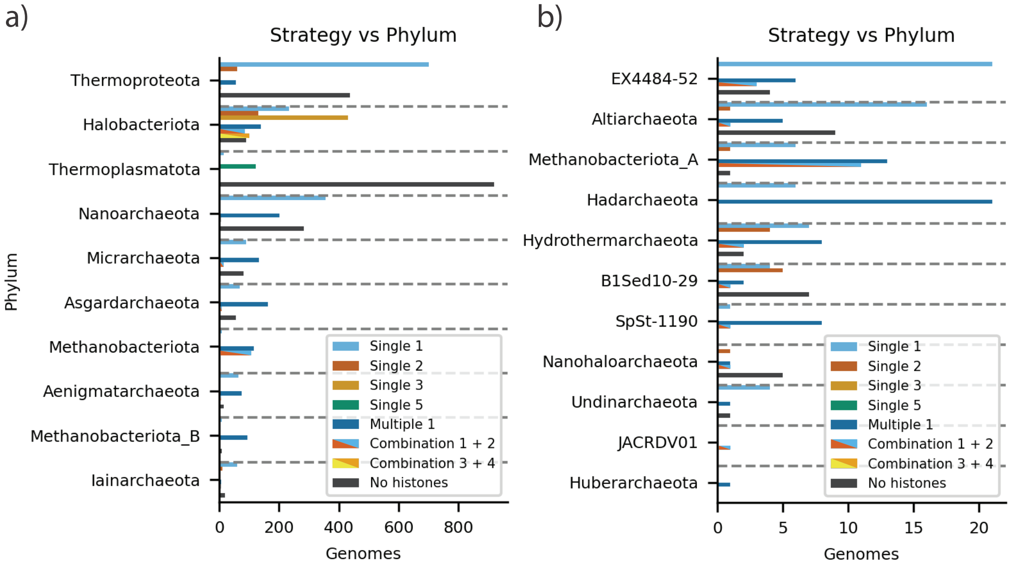

Supplementary Figure 3: Number of genomes from archaeal phyla across employing a particular histone strategy. a) phyla which are represented by a large number of genomes. **b)** phyla with more sparse representation (note difference x-axes scale).

Supplementary Figure 4

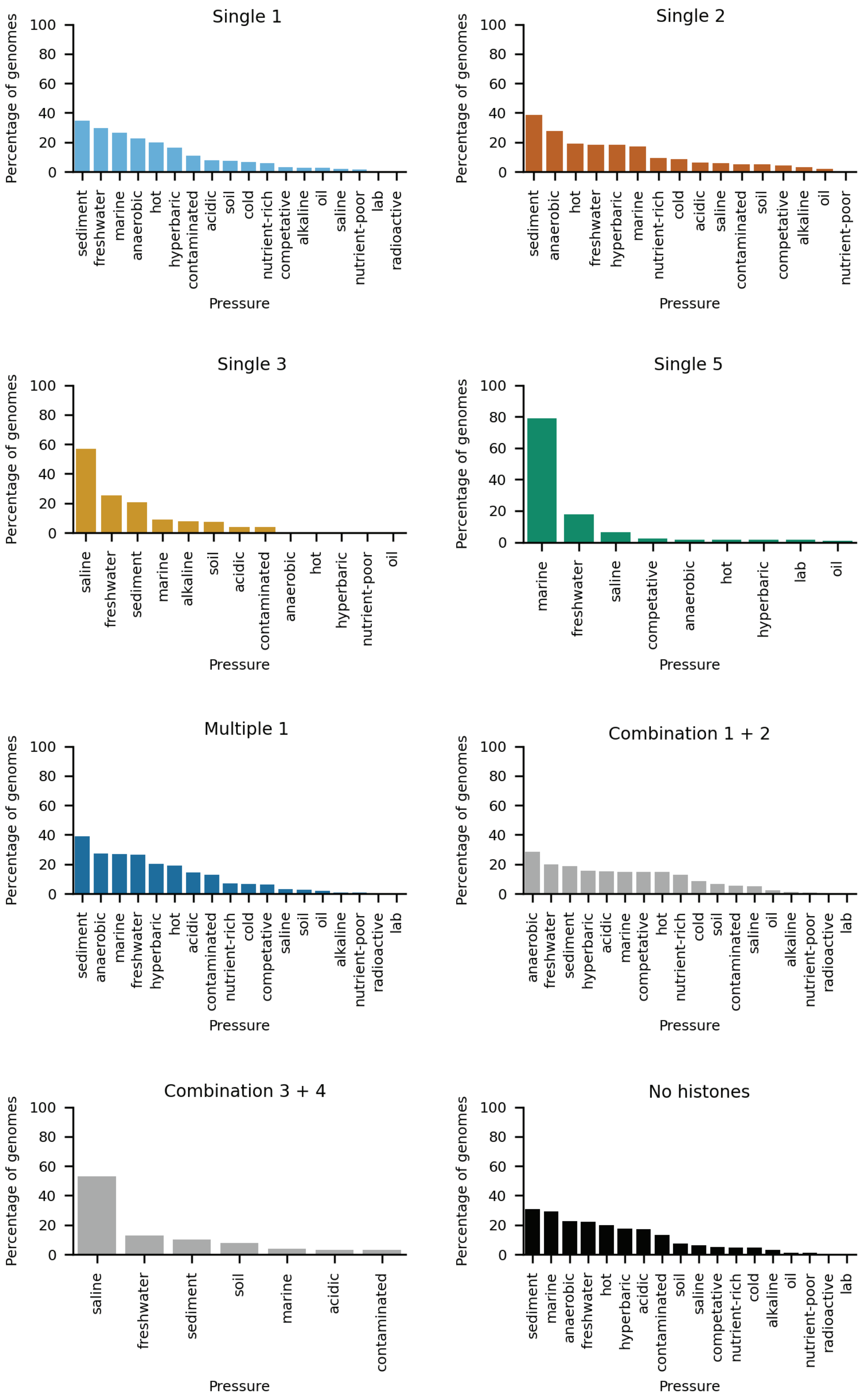

Supplementary Figure 4: Histone strategy correlates with environmental pressure. Sampling locations for each genome in a strategy were curated from metadata and coded to common environmental pressures, then plotted as the percentage of genomes from that strategy that are associated with that pressure. Locations can be associated with multiple pressures. Pressures are ranked from most prevalent to least. Single 3 and Combination 3&4 showed a slight bias towards genomes from saline environments. Single 5 genomes are biased towards marine environments. Combination 1&2 genomes correlate with anaerobic environments.

Supplementary Figure 5

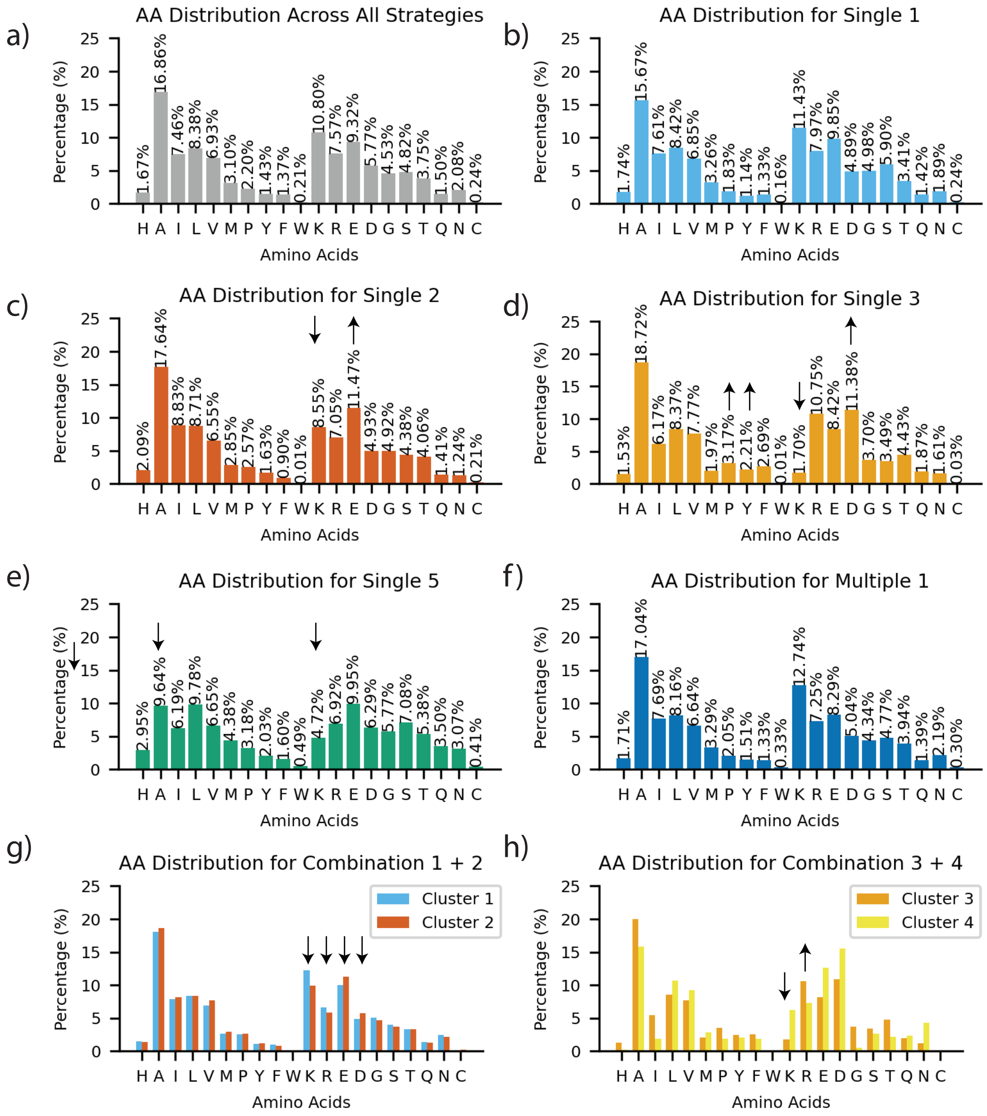

Supplementary Figure 5: Amino acid composition of histones, grouped by strategy. Contribution of each histone is normalized for length, so that larger proteins do not dominate the average composition. Overall, archaeal histones are enriched in small, hydrophobic residues and basic residues. Tryptophan and cysteine are rarer compared to the 'universal' proteome, likely due to their metabolic burden. Arrows denote enrichment or depletion of a particular genome compared to the generic archaeal amino acid distribution. **a)** All archaeal histones; **b)** basic singlets (single 1); **c)** acidic singlet (single 2), arrows denote the shift in composition from lysine to glutamate, responsible for their acidic character. **d)** Acidic doublets (single 3). Arrows denote the increase of aromatic residues tyrosine and phenylalanine, as well as a marked shift away from lysine in favor of arginine and an enrichment in aspartate over glutamate. **e)** Single 5 histones,

arrows denote the decrease in abundance of alanine and lysine. **f)** Multiple cluster 1 histones. **g)** Combination 1&2 histones. Arrows indicate the increase of basic and reduction of acidic residues in cluster 1 histones (blue) over cluster 2 histones (red). **h)** Combination 3&4 histones. Arrows indicate the increase of arginines and decrease of lysines in cluster 3 histones over cluster 4 histones.

Supplementary Figure 6

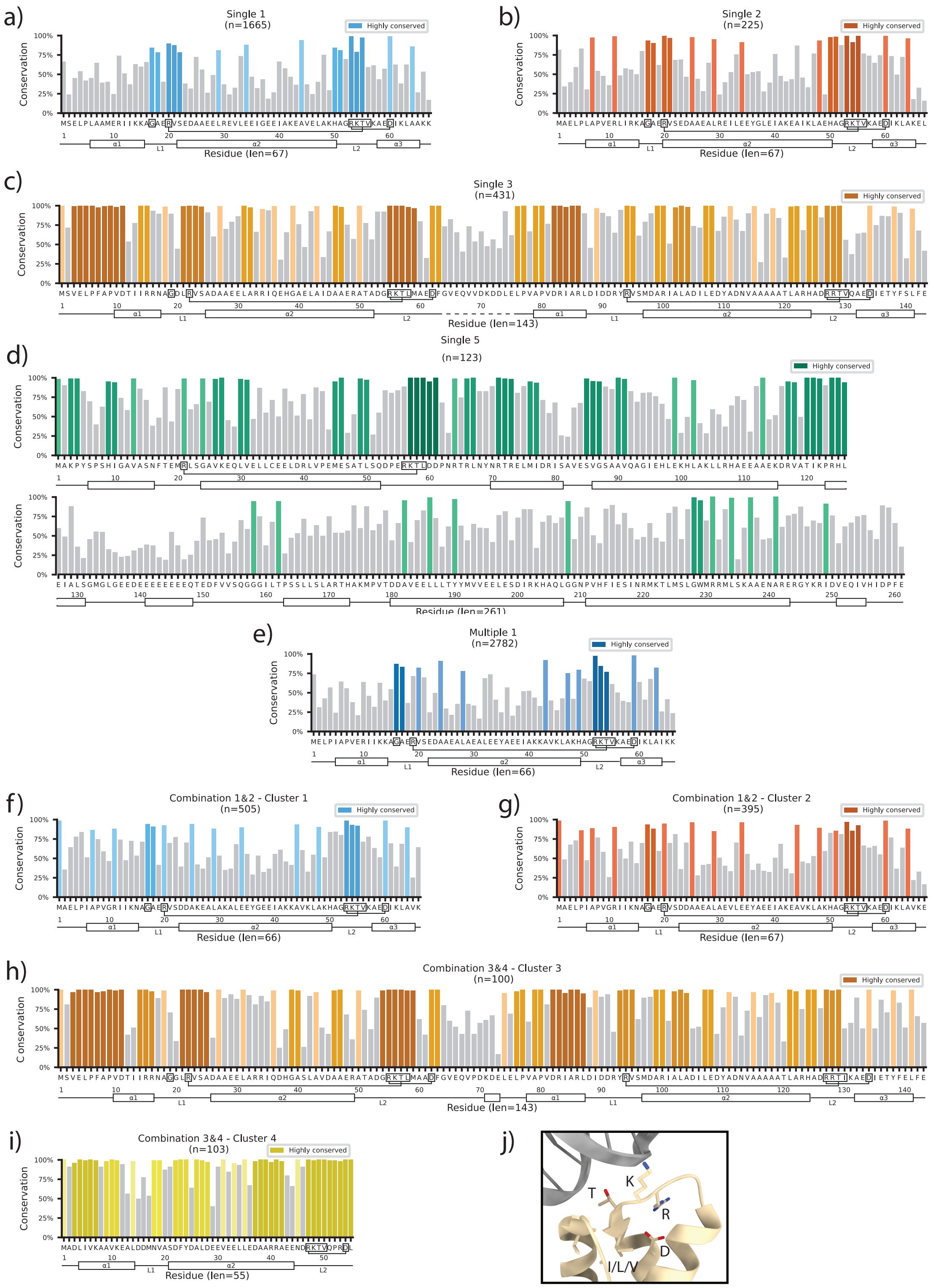

Supplementary Figure 6: Conservation of histones by strategy. Amino acids that are conserved by more than one standard deviation than the mean conservation of an alignment are highlighted in color. Continuous clusters of residues having greater than one standard deviation of conversation are shaded in darker color for emphasis. Number of sequences in each alignment is denoted for each panel as "n=". The average alignment length is denoted by "len=". Conserved sequence motifs (RKTV motif, R-D clamp, RT pair, and G are boxed in the sequence, H-bonds for R-DNA clamp and RT pair are indicated). Predicted secondary structure designation (from Supplementary Figure 7) are shown to indicate histone fold elements. **a-d)** singles, **e-i)** combinations, **j)** shows the L1L2 loop with conserved features, as indicated in the sequence alignments (pdb 5T5K).

Supplementary Figure 7

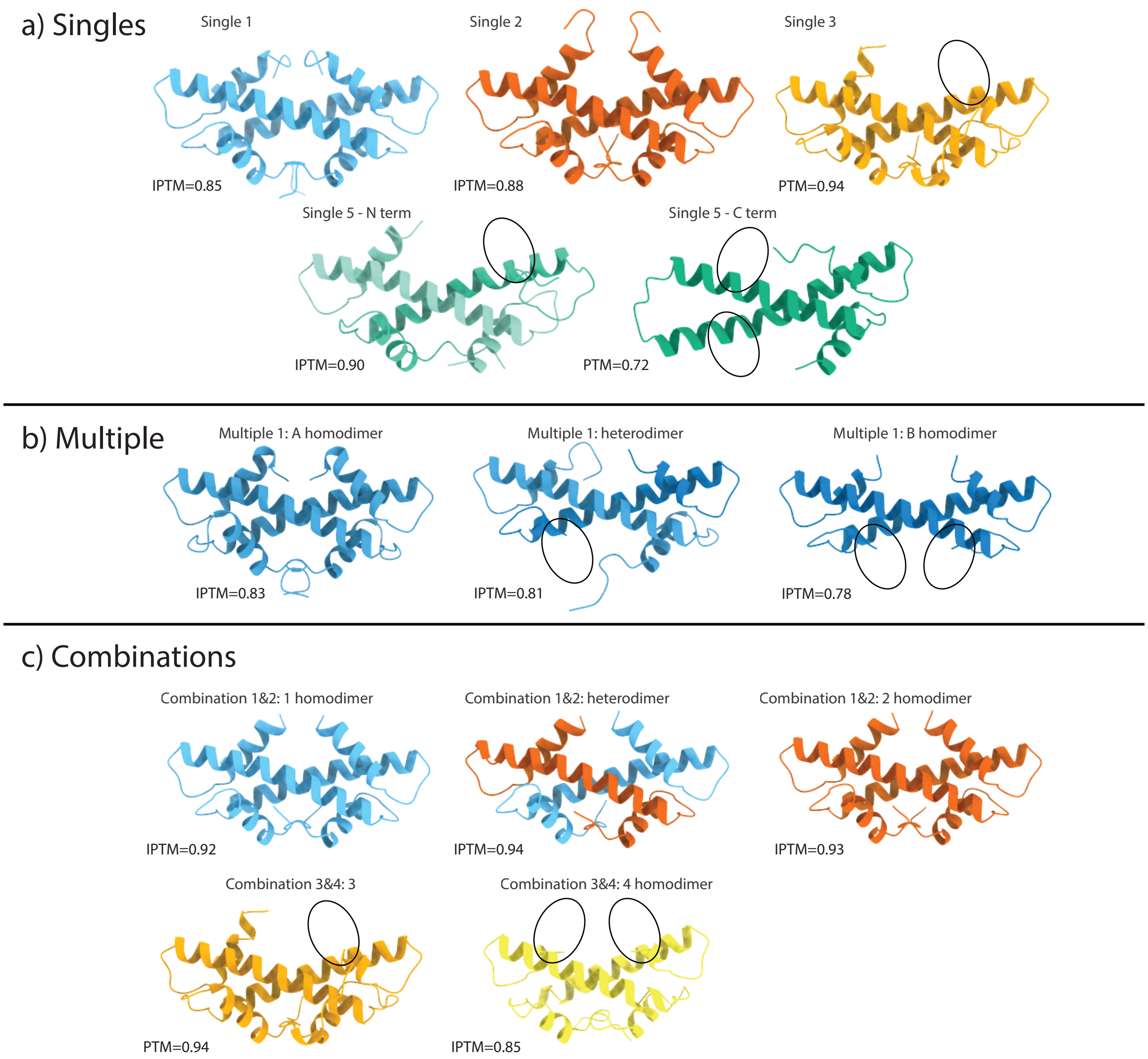

Supplementary Figure 7: AlphaFold3 predictions of histone dimers. Models predicted from representative ('median') histone fold domains from each histone strategy (Table 2). Helices missing from the classic three-helix histone fold motif are indicated by circles. **a)** Histone dimers from representative organisms with only a single histone gene. For the single 5 histone, which contains five histone fold domains, the N-terminal two histone folds are split into separate chains and predicted as if belonging to two separate chains, whereas the C-terminal two folds were predicted as a single chain. **b)** Prediction of homo- and heterodimer histone fold structures from a representative of the multiple 1 strategy. **c)** Combination 1&2**:** basic and acidic histones can form

homo- and heterodimers. Charged surface representation of the histone binding ridge are shown to the side of each homodimer Combination 3&4 histones were not folded together, as cluster 3 histones links two histone folds together in one polypeptide chain. All predictions have a high confidence score (IPTM).

Supplementary Figure 8

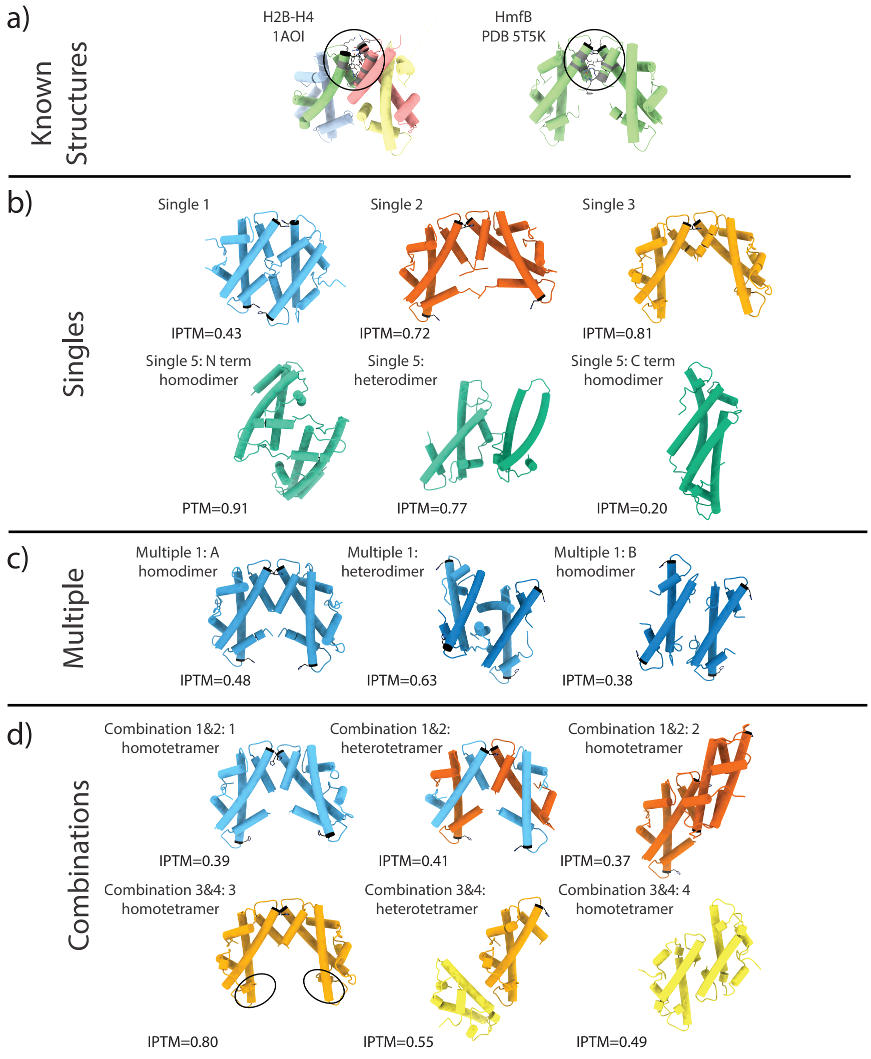

Supplementary Figure 8: AlphaFold3 prediction of representative histone tetramers. Models predicted from four histone fold domains for the median sequence of each histone strategy. a) Four-helix bundle structures from experimentally determined structures (not predicted through AlphaFold3). The conserved histidine is shown in black. **b)** Predicted homo-tetramers from single histone strategies. The Single 1 histone can form either closed (as shown here) or open tetramers with similar levels of confidence. Tetramers from the Cluster 5 histone do not appear to form canonical histone tetramers via four-helix bundle structures. **c)** Homo-tetramers from the multiple 1 genome vary in their predicted ability to form canonical open histone tetramers. **d)** The acidic histone from combination 1&2 forms canonical histone tetramers if paired with its basic partner. In isolation, it is predicted to fold into four completely different tetramers with similar confidence (inset; α3 helices are colored in grey and yellow for histone fold dimer 1 and 2, respectively; for

orientation). The cluster 3 histone from combination 3&4 in isolation forms an open tetramer, but does not combine with its cluster 4 partner. The cluster 4 histone in isolation is predicted to form ‘back-to-back’ structures (shown), as well as closed tetramers with similar confidence.

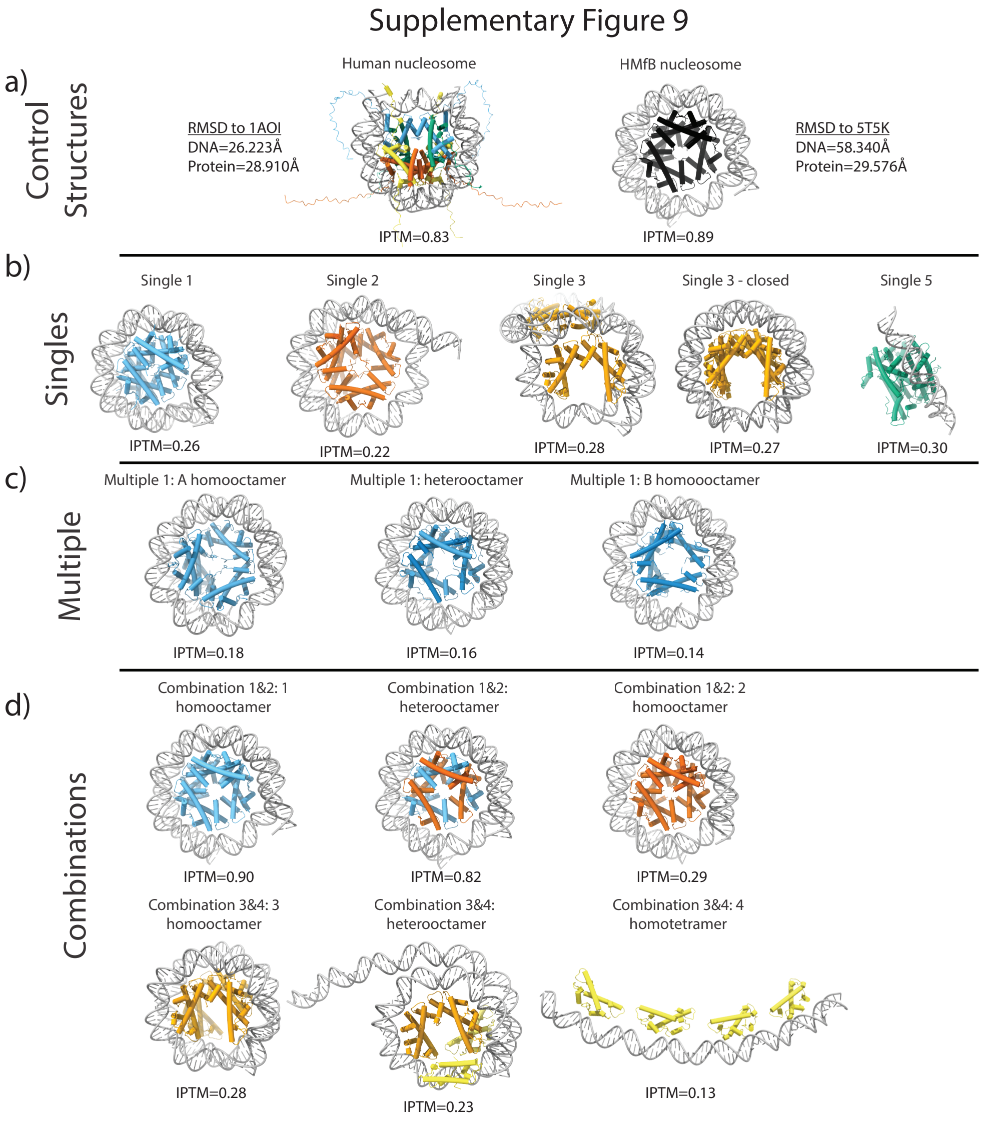

Supplementary Figure 9: Predicting histone-DNA complexes with AlphaFold3. Models predicted from eight histone fold domains for a representative from each histone strategy, and 147bp of a nucleosome positioning sequence ('601' Widom DNA sequence). These serve as the starting structures for the simulations shown in Figure 5. **a)** AlphaFold3 prediction of control structures of human and *M. fervidus* nucleosomes, closely resembling experimentally determined structures. **b)** Predictions for a basic and acidic singlet, and for the acidic doublet. For single 3, a second structure was predicted of a closely related histone (NZ_A0AIB010000141_204) that formed a closed nucleosome structure. **c)** the representative histones from the multiple 1 strategy appear to form nucleosome-like particles in each combination. **d)** Basic and acidic histones that

co-exist in one genome fold into nucleosome-like structures either individually or in combination. In contrast, the acidic doublet does not combine with the acidic miniature, which on its own does not wrap DNA, nor does single 5.

Supplementary Figure 10

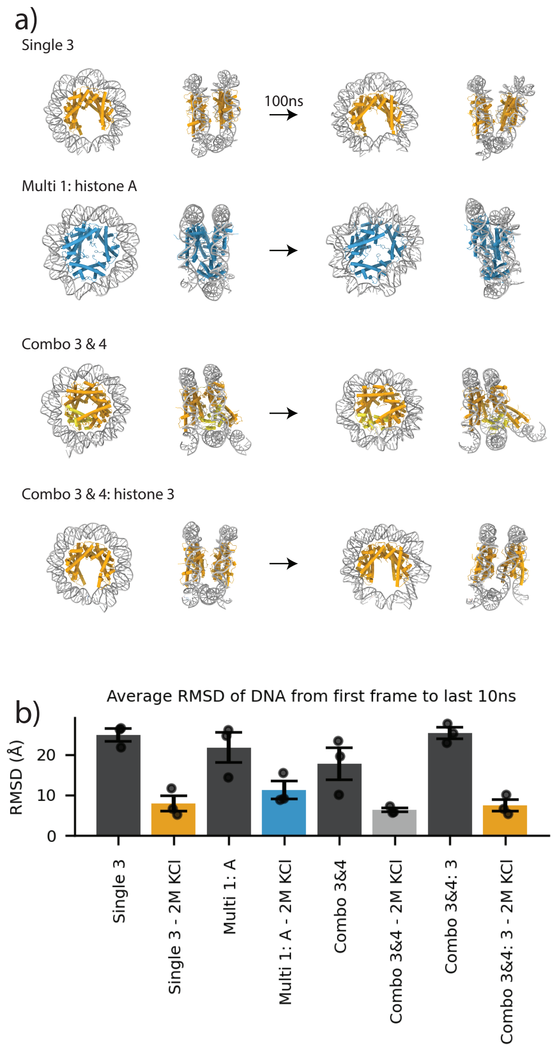

Supplementary Figure 10: Some nucleosomes may be more stable in high salt conditions. Several nucleosome-like structure that were unstable after 100 ns of simulation were simulated for 100 ns in the presence of 2 M KCl. This increased their apparent stability. As three of the four histones (Single 3, Multiple 1: histone A, Combination 3&4, and Combination 3&4: histone 3) are encoded by predominately halophilic organisms, we reasoned that higher ionic strength might be necessary for structural stability. **a)** Snapshots of starting and ending structures from one representative replicate simulation in both face and side views. **b)** Changes in DNA topology from beginning to end of simulations compared to the original (neutralizing KCl) simulations. RMSDs (Å) were calculated by averaging the RMSD of the DNA in each structure over the last 10 ns of the simulation to the starting frame in three replicate simulations.

Supplemental Figure 11

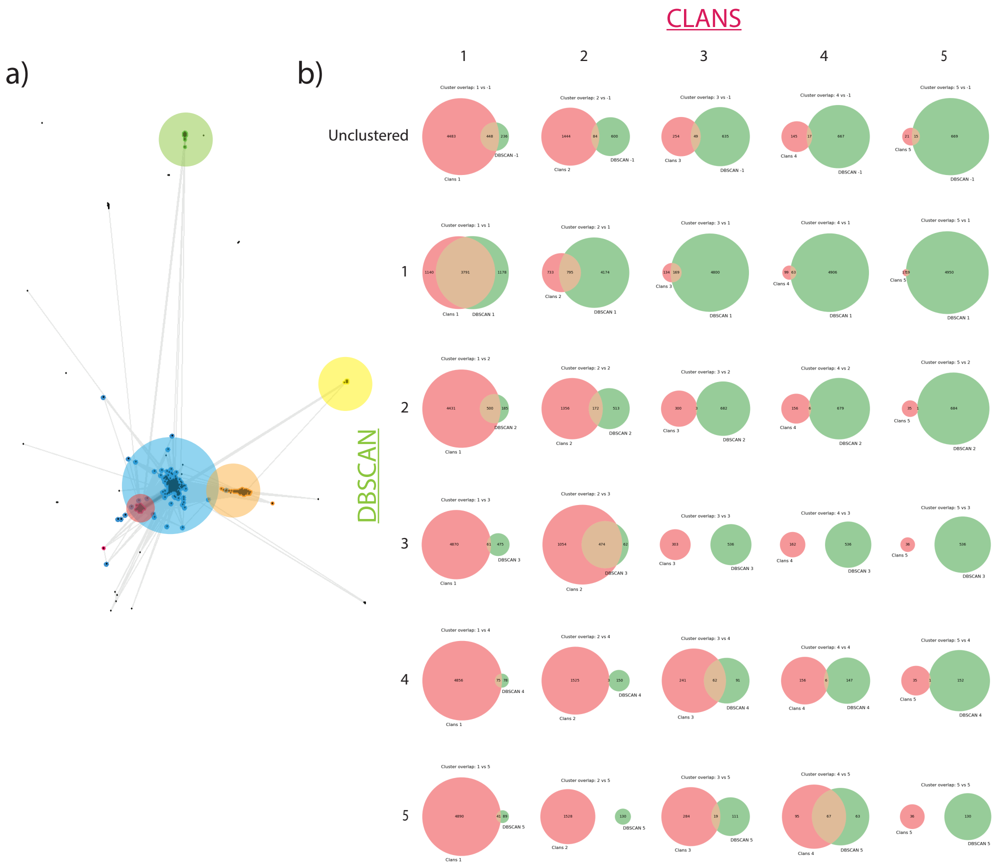

Supplementary Figure 11: Overlap between CLANS and DBSCAN clustering. Clustering was repeated on the GTDB histone hits using similar methods as in Schwab et al. (reference 14). Briefly, histones were aligned using the CLANS online toolkit. Sequences were then clustered using the CLANS Java application by clustering for 40,000 rounds. As in reference 14, the minimal attraction parameter was set to 50, while all other parameters were left as default. Clusters were assigned using the “Network based” algorithm in CLANS using 25 minimum sequences per cluster, with both “offset values” and “global average” enabled, for 100 rounds. **a)** Graphical representation of histone clusters. Individual dot (proteins) are labeled with cluster colors (blue: cluster 1, red: cluster 2, orange: cluster 3, yellow: cluster 4, green: cluster 5). Additional circles are used to highlight regions containing those clusters. **b)** Venn diagrams comparing CLANS labeled clusters to DBSCAN labeled clustering. There is only limited agreement between CLANS (in red) and DBSCAN clustering (in green).

Supplemental Figure 12

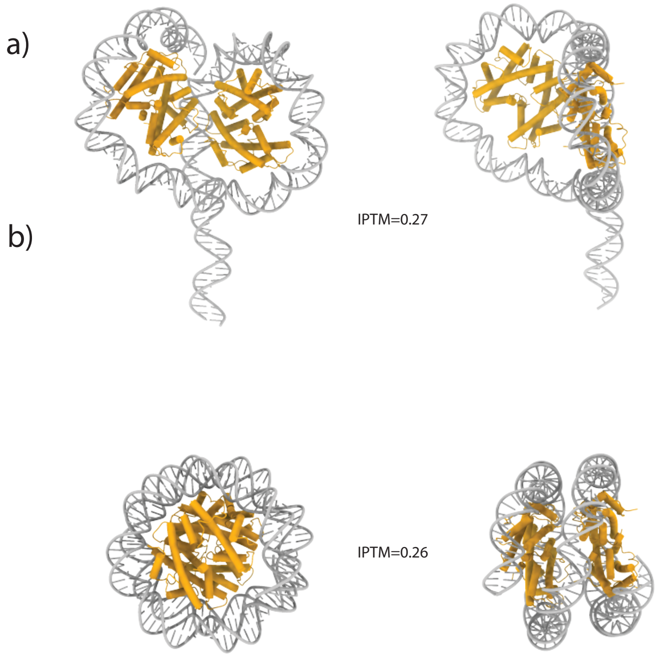

Supplementary Figure 12: Alphafold prediction of *Methanopyrus kandleri* histone with 147 base pairs of DNA. AlphaFold3 prediction of the doublet histone NC_003551.1_1799 from *Methanopyrus kandleri* (this organism also contains two Cluster 1 histones). Most of the predictions of this structure suggest flexible or unstable nucleosome conformations (11 out of 15 models). More plausible histone-DNA structures in this organism may require either one or both of the cluster 1 histones also present in the genome, or additional protein cofactors. a) Side and face view of one representative open prediction, suggesting the instability of a nucleosome formed with four doublet histones. **b)** one of the few models from AlphaFold displaying a closed configuration, suggesting at least the possibility of nucleosome-like structures with this acidic doublet.

Supplemental Figure 13

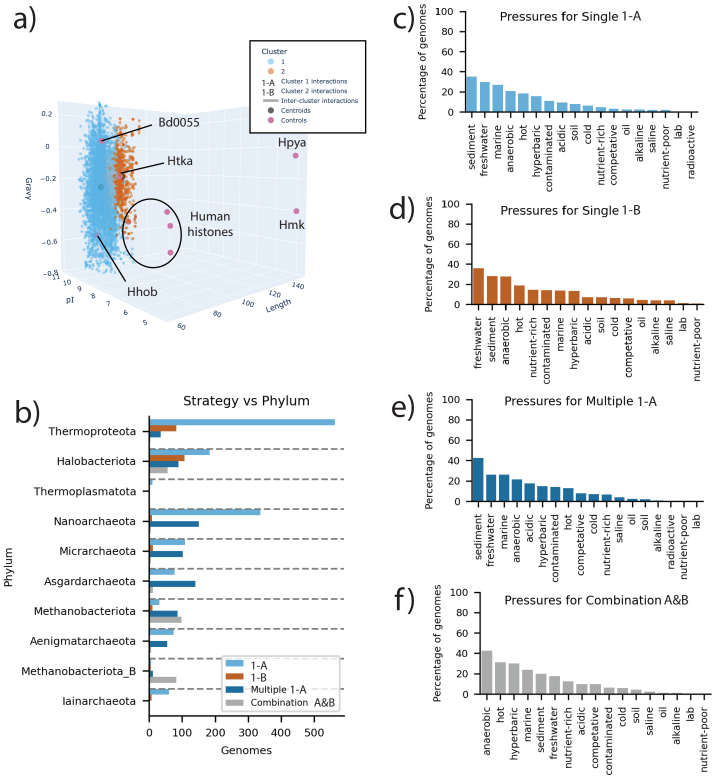

Supplementary Figure 13: Re-clustering of cluster 1 histones. Histones from cluster 1 were re-clustered using the same methods. This results in the appearance of two sub-clusters within Cluster 1 (sub-cluster 1-A and 1-B) which differ primarily in isoelectric point. **a)** Clustering of histones plotted by sequence length (length), isoelectric point (pI), and hydrophobicity (GRAVY). Representative known histones (human histones H2A, H2B, H3, and H4; archaeal histones Htka, Hhob, Hmk and Hpya; and bacterial histones Bd0055) are also plotted. **b)** Number of histones in each cluster from panel a, found in specific phyla. **c-f)** Rank of selective pressures of the genomes containing **c)** Single 1-A, **d)** Single 1-B, **e)** Multiple 1-A, and **f)** Combination 1-A and 1-B.
