# Supplementary material for: Histone diversity in the archaeal domain of life
Source: Nat Commun. 2026 Apr 15;17:5254. doi: 10.1038/s41467-026-71849-3 (PMC13260811; doi:10.1038/s41467-026-71849-3)
Supplement: Supplementary file 3 — Transparent Peer Review file [file 41467_2026_71849_MOESM3_ESM.pdf]

nature portfolio

Peer Review File

Histone diversity in the archaeal domain of life

Corresponding Author: Professor Karolin Luger

This file contains all reviewer reports in order by version, followed by all author rebuttals in order by version.

Version 0:

Reviewer comments:

Reviewer #1

(Remarks to the Author)
The paper by Laursen and Luger "Histone diversity in the archaeal domain of life" aims to characterize the diversity of histone-fold-containing proteins across the Archaea. This is done using their protein sequences and with bioinformatics tools: finding archaeal proteins containing the histone fold, clustering them based on basic structural properties, and analyzing representative proteins from each cluster based on their taxonomic distribution and predicted binding characteristics.
The extensive use of bioinformatics tools including Alphafold3 and molecular dynamics is an innovative approach and allows the analysis of thousands of proteins from species that have not been cultured. Clustering by length and pI gives a useful guide to the diversity of archaeal histone proteins, there are interesting insights from the structural predictions, and the limitations of these predictions are acknowledged. This study therefore has the potential to be an important resource for the archaeal research community.
However, additional clarity, expansion on what is gained from these data before the resource is accessible to the community. Importantly, more thorough treatment comparing their results to existing evidence in the field on archaeal histones are needed to test and somehow benchmark the reported predictions. Specific comments are given below.
Major issues/suggestions:
1. There are 5 clusters, however, cluster I contains ~60% of all analysed sequences. This is an important point that should be emphasized in the text, not just mentioned in Table 1. This result is also relevant to a few more suggestions/issues mentioned below.
2. Cluster 5 histones seem to be quite diverged in sequence and possible function from other archaeal histones. There are histone-fold proteins outside archaea, like the NF-Y transcription factor family, which are not involved in nucleosome formation. Would it be useful to add them to the proteins being clustered, and might they have any relation to cluster 5?
3. Eukaryotic histones are always basic, and all basic archaeal histones are within cluster I. This cluster contains a few highly conserved and many less constrained residues (Fig S6). It is also present across the vast majority of archaeal taxa (Fig 3). This cluster contains the best-characterized archaeal histones, with the best experimental evidence for nucleosome formation. For all these reasons, I believe it is worth performing a clustering within this cluster, to see if there are significant differences within these "canonical" or "typical" archaeal histones, and how these possible differences relate to single- or multi-histone strategy, phylogeny, and growth environment (see below).
4. Previous studies (Hocher et al 2022) suggest a link between growth temperature and chromatinization in Archaea (using both histone and non-histone proteins). When considering all archaeal histones, no link between "hot" environment and histone cluster was found (Fig 4). However, this might be different when looking at sub-clusters of cluster I as suggested in point 3 above. Can the authors try this to determine if they can recapitulate this correlation that has been previously observed?
5. In silico DNA binding studies were done using the eukaryotic nucleosome length of 147bp. However, MNase data from various species usually suggests that archaeal histones typically bind DNA in ~60bp increments. The authors should try differing MNase digest lengths to make the simulations more physiologically relevant to what is already known about some archaeal histones.
6. Additional results section & a figure (and/or experiments) are needed to test how the predictions align (or conflict) with experimental data and discuss possible reasons for alignment or discrepancy. For example, apart from M. fervidus and T. kodakarensis, both mentioned in the introduction, an archaeal histone that has been shown to form nucleosomes in vitro is the doublet histone of Methanopyrus kandleri, whose structure has been characterized by x-ray crystallography (Pavlov 2002 J Biomol Struct Dyn, Fahrner 2008, Moudrianakas and colleagues). Which cluster does M. kandleri histone fall in, and what do MD and Alphafold predict about its dimerization and DNA binding? Does this match previous experimental observations? Similarly, Thermococcales histones have been characterized by structural, functional genomics, and genetics

(see work of Santangelo, Reeve, and colleagues). Doublets from haloarchaea and methanogens have also been characterized by functional genomics and other methods, showing that they likely do not package DNA (Dulmage 2015, Sakrikar 2021 and 2023, Weidenbach et al., 2008, etc). How do the authors reconcile these prior findings with the alphafold predictions that all the histones studied likely wrap DNA? How does a protein with an acidic PI wrap DNA? Indeed, the authors mention in the discussion that, “a logical next step to this work is to use structural biology and biochemistry to uncover how these histones physically structure DNA…” a lot of work (in addition to other experimental methods to characterize histones) has been done already in archaea - comparing their predictions with these data are important controls to validate the predictions, especially when dealing with relatively understudied variants of histones that the AI-based systems like alphafold may not been trained on. Can the authors add a section to the results that expounds on such a comparison?

1. Are pI predictions normalized to the distribution of the database? The claim that “overall, a surprisingly large percentage (26.7 %) of the 7,157 predicted histones are acidic in character”. may not be is this surprising if the database is dominated by haloarchaea or other salt-in strategists, for example. As the authors note elsewhere in the manuscript, these organisms have overall acidic proteomes, and because of historical reasons for ease of culturing, tend to be overrepresented in genome databases. Please normalize / clarify whether acidity of histones is significantly enriched relative to the background distribution of proteomes in the database (for example, by Fisher’s exact test or hypergeometric test). Related, the clusters that contain the acidic doublets seem larger than other clusters, and all known haloarchael genomes encode the acidic doublet.
2. What is encoded nearby the various histone types? If a genome encodes histones as predicted monocistrons vs operons (and if in operons, what is the function of these genes?), this is important information that would enhance the analysis. Does genome neighborhood / synteny vary within clusters, or is it conserved? This is important evolutionary information. Such an analysis could enhance the predictions made.
3. Some histone genes seem to belong to two categories. For example, in haloarchaea, in most cases, the acidic doublet “combination 3&4” and “single 3” type histones seem to map to the same gene (encoding the acidic doublet) – are there any cases where “single 3” is encoded in a gene by itself (i.e. not fused to 4)? Is this purposeful, or an error? Or do protein categories not correspond to encoding genes per se? Please correct or clarify how “strategies” (histone categories / clusters) are defined throughout. Answering this question would be easily addressed by adding a column of gene identifiers to the supplementary tables that correspond to the proteins (together with protein identifiers that exist in commonly used databases, see minor comments below) so that users could look them up.

Minor comments:
1. For ease of comprehension, either the centroid histone or the best-studied histone from each cluster could be mentioned in the text, so that readers can put a “face” to a (cluster) name.
2. “Closed tetramer” is undefined (line 253 and throughout).
3. The results regarding Histones A and B from alphafold and MD appear to contradict (lines 264, 308). Can the authors expand on an explanation for this? Also, it would be better for clarity to explain what histone A and histone B are, before discussing these results.
4. Line 126: in the section “histones are used in 7 different strategies” – the phrase “acidic singlet (cluster 3)” is a typo, should probably read “acidic doublet”.
5. Please check the references throughout. There are some incorrect citations detected, especially in the intro, for example Line 69: Neither ref 32 nor 33 report ChIP-Seq data. Please reference Sakrikar et al., 2021 (https://doi.org/10.1093/nar/gkab1175) and 2023 (https://doi.org/10.1128/mbio.03449-22) for ChIP-seq with archaeal histones, and correct the text to say “…and ChIP-seq across haloarchaeal species…” (or add the other species that has been studied, i.e. Haloferax volcanii).
6. To me, the language of “histone strategy” comes across as anthropomorphic while simultaneously being unclear whether the authors refer to a prediction or an experimentally tested mechanism. “Strategy” is used throughout together with verbs that describe actions of organisms, which should be clarified to ensure that the reader understands that everything is coming from predictions (e.g. introduction, “strategies’ by which “organisms combine histones”; results line 113 section header “Histones are used…”; most of the figures…). I suggest that the authors use another descriptor to refer to the various combinations of histones encoded that is more aligned with what data the study is actually reporting. Example alternatives: “Seven different histone sets / groups / predicted DNA-protein complexes / types / categories are encoded in genomes”? There are many options that I think may more accurately refer to what they’re reporting. Please amend the language throughout to avoid overstating claims.
7. Line 119 “one single” is redundant, should be “one” or “a single”, please revise.
8. Protein and species identifier types seem to be inconsistent across ontologies in the supplementary data (RefSeq GCF ID’s and GenBank GCA), different database protein identifier types (protein identifiers – what database are those from? Some are not detected by any searches on databases including GenBank, UniProt, EMBL, etc…). Related, how are the supplementary organism identifiers organized (all RefSeq IDs for all species and strains included, or just type strains / multispecies GenBank IDs that combines all strains of a species? If just type strains, why and how were these selected)? type species and / or species with experimentally characterized histones are missing in some cases. Some more careful checking and explanation of these supplementary files would be helpful to the community, and may lead to wider use of the dataset.

Reviewer #3

(Remarks to the Author)
The manuscript “Histone diversity in the archaeal domain of life” by Laursen and Luger describes an in silico structural study of archaeal histones. First, all available archaeal genomes are searched for histone genes and 67% of known archaea

contain histone genes. These histone genes were grouped in five clusters based on physical parameters and subsequently for each genome, the histone gene diversity was determined, and these were found across the whole archaeal phylogeny tree. Most genomes only contained one histone gene or multiple from the same cluster. Delving deeper into the structural features of these histone clusters using AlphaFold3, the predicted use of the four-helix bundle to form a tetramer was assessed as well as the presence of histone signature motifs in loop1 and loop2. Finally, the capability of these archaeal tetramers to form stable association with DNA was predicted and this was subsequently tested by MD simulations. As a final note, the authors state that "predictions are not yet ready to stand on their own without experimental validation." Overall, it is a clearly written manuscript with easy-to-follow figures. This study is of interest to the broader chromosome field by providing a structural = view of histone evolution in archaeal species. This reviewer only has a few concerns.

Major concerns:
+ The various archaeal histone genes were clustered based on physical parameters. Traditionally, genes are aligned and evolutionary relationship depicted in a phylogeny tree, and the accuracy of their relationship is tested by bootstrapping. If this latter approach is used, would this provide the same 5 groups of histones?
+ On page 9, tetramer formation for multiple 1 and combination 1&2 are discussed, but it is unclear if four-helix bundle is formed in these cases based on the AlphaFold3 predictions. Please clarify this.
+ The cartoon for Supplemental Figure 6 is tricky to follow. A tetramer where loop 1 and loop 2 are highlighted would be more informative.
+ For the known structures in Supplemental Figure 8, were these also run in AlphaFold3 as controls? This is unclear from the figure and text.

Minor concerns:
+ Figure 1b, it would be informative if the percentage of the total genes is reported per cluster.
+ page 7, line 180 "… much divergent …" should be "… much more divergent …".
+ One page 7 line 209 to 8 line 211, a sentence in the middle of a paragraph is in bold.
+ The term "four-helix bundle" is abbreviated 4HB, but the term is only used seven times in total. Writing out four-helix bundle might be more accessible to non-histone aficionados.

Reviewer #4

(Remarks to the Author)
I co-reviewed this manuscript with one of the reviewers who provided the listed reports. This is part of the Nature Communications initiative to facilitate training in peer review and to provide appropriate recognition for Early Career Researchers who co-review manuscripts.

Reviewer #5

(Remarks to the Author)
The Authors provide in depth in silico analyses of the histone family in Archaea and the interactions of these proteins with DNA. Based on structure prediction, simulations and their in depth expertise in structures of the nucleosome(s), the survey yield five groups of histones based on their predicted structure and physical parameters. Groups 1 and 2 contain the typical histone fold typical of the well studied HMfB histone. Group 3 comprise two histone fold domain linked, Group 4 comprise short acidic proteins with truncation of helices 2 and 3 and group 5 contain four linked histone folds but lack many classic histone features and might have roles other than histones. There is no strong correlation of the presence of specific groups based on phylogeny, genome size, GC content and ecology, although as noted by the Authors, the paucity of informations on ecology and physiology limit this type of analysis. Importantly most archaeal histone are more acidic than their eukaryotic counterparts (exception group 1) and they show a universal conservation of residues in the L1 and L2 loops and groups 1 to 3 have the capacity to form homodimers but also heterodimers in genome encoding different types of histones 1 and 2 and even in the combination of group 3 and shorter histones of group 4. Various possible tetramers assembling two dimers on DNA are also evaluated with limitations of alphafold 3 overcome by molecular dynamics simulations indicating the likelihood of each combination.
Overall the study of of high interest for a broad and well written for a broad readership. There are only a series of minor comments that need to be addressed.

1. Fig2 illustrate that most genome contain a single type of histone. However the majority of genomes used in the study are not complete and originate from metagenomics and have not been manually curated. Hence it is likely that some genes including histone gene might be missing or that histones from groups 3 to 5 might have not been recognised as bona fide proteins and been removed from the genomes. So it would be important that the Authors remain caution about their conclusions. in addition, a very few species of archaea have been identified through cultivation while most remain "candidatus xxx". So the Authors should refer to genomes rather than species.

2.Fig5b is crucial in the study and as pointed by the Author should be interpreted with perhaps more caution. Single 3 strategies look not so stable and it would be good that the Authors provide the simulations at 2KCl.

3. line 340 . As far as we understand, it is currently not possible to trace the similarity of archaeal histone to each type of eukaryotic histone. So it is logically not possible to directly compare the diversification from archaea versus eukaryotes in these terms. The Authors might rather state that as eukaryotes, archaea diversified their histones into subtypes.

4. As mentioned by the Authors, a broad survey of archaeal histone has been recently published based on in part similar criteria ref 14. It would be of high interest to the readers that the Authors compare the two studies in a Table showing overlap of the two classifications.

5.SupFig6 b the extension of the alpha2 helix might be erroneous because it overlaps with the RV motif normally in the L1. Fig2i should be placed in position d and line 206 likely refers to the current pnal j, which should be also mentioned in line 203.

6. line 317 typo on strength.

Version 1:

Reviewer comments:

Reviewer #1

(Remarks to the Author)
We thank the author for the thorough consideration and response to our previous review. The previous concerns have mostly been addressed. One remaining concern still needs to be addressed (#5):

5. Reviewer comment: In silico DNA binding studies were done using the eukaryotic nucleosome length of 147bp. However, MNase data from various species usually suggests that archaeal histones typically bind DNA in ~60bp increments. The authors should try differing MNase digest lengths to make the simulations more physiologically relevant to what is already known about some archaeal histones.
Author reply; We were explicitly looking for hypernucleosome structures, and therefore limited our simulations to 147 bp.

Can the authors clarify / justify in the main text why they used 147bp DNA in their AlphaFold structural predictions of histone-DNA interactions? The authors state in the introduction that 147bp is the length known to wrap eukaryotic nucleosomes, but their own structural work (Mattrioli et al., 2017) , in vivo MNase-seq of others (Ammar et al. 2012; Maryuama 2013) , in vitro studies from Reeve's group have all shown that the archaeal nucleosome wraps less DNA (for example, in Mattrioli et al, 90 bp for one nucleosome, 120bp for multiple stacked hypernucleosomes, sometimes less DNA in MNase-seq). I understand that the authors were explicitly looking for hypernucleosome structures and therefore I understand the need to use more than 60 bp in their structural precitions. But why did they use only one length of DNA with such a specifically eukaryotic bias (and not 60bp, 90bp, or other multiples thereof)? As previously requested, can the authors either provide a stronger justification for using 147bp and/or conduct the structural predictions with a more physiologically relevant DNA length?

(Remarks on code availability)

Reviewer #3

(Remarks to the Author)
The authors have addressed all concerns raised by this reviewer and has no further concerns. This reviewer believes the work is ready for publication.

(Remarks on code availability)

Reviewer #5

(Remarks to the Author)
The Authors have addressed the concerns raised by all reviewers and the resulting revised manuscript is much improved and ready to be published.

(Remarks on code availability)

Version 2:

Reviewer comments:

Reviewer #1

(Remarks to the Author)
Thank you for addressing my previous comment about the choice of 147bp for the structural modeling of the archaeal nucleosome. Please include the rationale you provided for me in the manuscript (for example, in the methods section). All other comments have been sufficiently addressed.

(Remarks on code availability)

Open Access This Peer Review File is licensed under a Creative Commons Attribution 4.0 International License, which permits use, sharing, adaptation, distribution and reproduction in any medium or format, as long as you give appropriate credit to the original author(s) and the source, provide a link to the Creative Commons license, and indicate if changes were made.
In cases where reviewers are anonymous, credit should be given to 'Anonymous Referee' and the source.
The images or other third party material in this Peer Review File are included in the article’s Creative Commons license, unless indicated otherwise in a credit line to the material. If material is not included in the article’s Creative Commons license and your intended use is not permitted by statutory regulation or exceeds the permitted use, you will need to obtain permission directly from the copyright holder.
To view a copy of this license, visit https://creativecommons.org/licenses/by/4.0/

REVIEWER COMMENTS

Reviewer #1 (Remarks to the Author):

The paper by Laursen and Luger "Histone diversity in the archaeal domain of life" aims to characterize the diversity of histone-fold-containing proteins across the Archaea. This is done using their protein sequences and with bioinformatics tools: finding archaeal proteins containing the histone fold, clustering them based on basic structural properties, and analyzing representative proteins from each cluster based on their taxonomic distribution and predicted binding characteristics.
The extensive use of bioinformatics tools including Alphafold3 and molecular dynamics is an innovative approach and allows the analysis of thousands of proteins from species that have not been cultured. Clustering by length and pI gives a useful guide to the diversity of archaeal histone proteins, there are interesting insights from the structural predictions, and the limitations of these predictions are acknowledged. This study therefore has the potential to be an important resource for the archaeal research community.
However, additional clarity, expansion on what is gained from these data before the resource is accessible to the community. Importantly, more thorough treatment comparing their results to existing evidence in the field on archaeal histones are needed to test and somehow benchmark the reported predictions. Specific comments are given below.

Thank you for your positive remarks.

Major issues/suggestions:

1. There are 5 clusters, however, cluster I contains ~60% of all analysed sequences. This is an important point that should be emphasized in the text, not just mentioned in Table 1. This result is also relevant to a few more suggestions/issues mentioned below.

We modified the statement (line 140): “Histones that fall into cluster 1 (basic singlets) are widely dispersed across the entire domain of archaea and seem to represent the typical archaeal histone, accounting for 77% percent of all clustered archaeal histones (Figure 3a).”
We also added percentages to Figure 1. Also see response to your point 3.

2. Cluster 5 histones seem to be quite diverged in sequence and possible function from other archaeal histones. There are histone-fold proteins outside archaea, like the NF-Y transcription factor family, which are not involved in nucleosome formation. Would it be useful to add them to the proteins being clustered, and might they have any relation to cluster 5?

The PFAM of archaeal histones (PFAM 808) used to identify histones through HMMsearches in this study include the NF-Y transcription factor family. It is a well established fact that the histone fold was co-opted by transcription factors (and in some cases as a protein-protein interaction motif without DNA binding capabilities).

3. Eukaryotic histones are always basic, and all basic archaeal histones are within cluster I. This cluster contains a few highly conserved and many less constrained residues (Fig S6). It is also present across the vast majority of archaeal taxa (Fig 3). This cluster contains the best-characterized archaeal histones, with the best experimental evidence for nucleosome formation. For all these reasons, I believe it is worth performing a clustering within this cluster, to see if there are significant differences within these “canonical” or “typical” archaeal histones, and how these possible differences relate to single- or multi-histone strategy, phylogeny, and growth environment (see below).

Thank you for this suggestion. We performed this clustering, presented in the new **Supplementary Figure 13**. We only pick up two prominent ‘sub-clusters’. This is already apparent in Figure 1, but much more obvious in the new clustering. The only real distinction between these clusters is the isoelectric point. One point of interest is that no genome contains multiple histones from the new 1-B sub-cluster, although they are found in combination with 1-A. The genomes with a combination of 1-A and 1-B seem to be more enriched in anaerobic and thermophilic environments. We have added text to lin 143.

4. Previous studies (Hocher et al 2022) suggest a link between growth temperature and chromatinization in Archaea (using both histone and non-histone proteins). When considering all archaeal histones, no link between “hot” environment and histone cluster was found (Fig 4). However, this might be different when looking at sub-clusters of cluster I as suggested in point 3 above. Can the authors try this to determine if they can recapitulate this correlation that has been previously observed?

It appears the “hot” environments are present in both halves of Cluster 1 histones, but that there could be an enrichment of “hot” environments in genomes which contain a histone from both subclusters of cluster 1 (see new Supplementary Figure 13f). As this is largely speculative, we decided against mentioning this in the revised manuscript.

5. In silico DNA binding studies were done using the eukaryotic nucleosome length of 147bp. However, MNase data from various species usually suggests that archaeal histones typically bind DNA in ~60bp increments. The authors should try differing MNase digest lengths to make the simulations more physiologically relevant to what is already known about some archaeal histones.
We were explicitly looking for hypernucleosome structures, and therefore limited our simulations to 147 bp.

6. Additional results section & a figure (and/or experiments) are needed to test how the predictions align (or conflict) with experimental data and discuss possible reasons for alignment or discrepancy. For example, apart from M. fervidus and T. kodakarensis, both mentioned in the introduction, an archaeal histone that has been shown to form nucleosomes in vitro is the doublet histone of Methanopyrus kandleri, whose structure has been characterized by x-ray crystallography (Pavlov 2002 J Biomol Struct Dyn, Fahrner 2008, Moudrianakas and colleagues). Which cluster does M. kandleri histone fall in, and what do MD and Alphafold predict about its dimerization and DNA binding? Does this match previous experimental observations?

We added text to line 108. We also performed alphafold predictions of four copies of the acidic doublet histone (cluster 3; NC_003551.1_1799) from *Methanopyrus kandleri* with 147 bp of DNA. Of the 20 predicted models, 15 were in an open, unstable configuration (representative shown in the new supplemental figure 12a) while 5 displayed a more closed configuration. Neither of these appeared very plausible, as also indicated by the very low IPTM scores. New text describing this finding has been added to line 297.

Similarly, Thermococcales histones have been characterized by structural, functional genomics, and genetics (see work of Santangelo, Reeve, and colleagues).

We are familiar with this work, and indeed references to the known structure are made throughout. However, the focus of this article is to identify new histone-like sequences, and not to analyze the effect of chromatin structure on genome regulation. We have added a statement acknowledging these studies to the discussion (line 399)

Doublets from haloarchaea and methanogens have also been characterized by functional genomics and other methods, showing that they likely do not package DNA (Dulmage 2015, Sakrikar 2021 and 2023, Weidenbach et al., 2008, etc). How do the authors reconcile these prior findings with the alphafold predictions that all the histones studied likely wrap DNA? How does a protein with an acidic PI wrap DNA? Indeed, the authors mention in the discussion that, “a logical next step to this work is to use structural biology and biochemistry to uncover how these histones physically structure DNA…” a lot of work (in addition to other experimental methods to characterize histones) has been done already in archaea - comparing their predictions with these data are important controls to validate the predictions, especially when dealing with relatively understudied variants of histones that the AI-based systems like alphafold may not been trained on. Can the authors add a section to the results that expounds on such a comparison?

The focus of our studies is to sample the breadth of archaeal histones in an agnostic way. Our analysis is not intended to imply that all archaeal histones are present on the genome in massive amounts, or that they are the main organizers of archaeal chromatin. We simply classify the various ways by which

archaeal histones oligomerize and potentially bind DNA. By and large, our findings are consistent with the few published functional studies on histones in select organisms which all demonstrate DNA binding, either directly or indirectly, and a role in transcription regulation. We allude to these publications wherever appropriate. For example, Dulmage et al. showed that HpyA, a cluster 3 histone, indeed appears to bind DNA in the cell. Although the authors point out that this histone might not compact the genome, the results are still consistent with our results. With respect to how acidic proteins bind DNA, this has been addressed in the literature (reference 44). We have added text to line 381. We have added a clarifying statement to the discussion (line 399): ‘ I*t must be emphasized however that a role for archaeal histones in genome compaction has not been firmly established even in the few model organisms that have been studied to date, and that histones might function as transcription factors instead’* (citation of the papers mentioned by this reviewer). *‘Answering this question requires the adoption of techniques commonly used to study genome organization in eukaryotes to the vast variety of archaeal organisms, many of which cannot even be cultured at this stage.’*

Reviewer 2:

1. Are pI predictions normalized to the distribution of the database? The claim that “overall, a surprisingly large percentage (26.7 %) of the 7,157 predicted histones are acidic in character”. may not be is this surprising if the database is dominated by haloarchaea or other salt-in strategists, for example. As the authors note elsewhere in the manuscript, these organisms have overall acidic proteomes, and because of historical reasons for ease of culturing, tend to be overrepresented in genome databases. Please normalize / clarify whether acidity of histones is significantly enriched relative to the background distribution of proteomes in the database (for example, by Fisher’s exact test or hypergeometric test). Related, the clusters that contain the acidic doublets seem larger than other clusters, and all known haloarchael genomes encode the acidic doublet.

It is not possible to normalize because the only genomes we know about are the ones that have been deposited. It is likely that halophiles are overrepresented to some extent, but we simply don’t know how many archaeal genomes / organisms remain to be discovered. We don’t know what we don’t know!

We added a statement at line 116. ‘*It is however possible that halophilic archaea are overrepresented in sequence space, and that the sequence distribution is currently skewed. As more and more archaeal genomes are published, the reported distributions are subject to change.*’

We are compelled to believe these proteins could function like histones because other halophilic (acidic) proteins are known to bind DNA, such as the transcription factor RosR.

2. What is encoded nearby the various histone types? If a genome encodes histones as predicted monocistrons vs operons (and if in operons, what is the function of these genes?), this is important information that would enhance the analysis. Does genome neighborhood / synteny vary within clusters, or is it conserved? This is important evolutionary information. Such an analysis could enhance the predictions made.

This would be a really interesting area to look into, however we believe it exceeds the scope of this manuscript and would be better left to a follow-up publication. We performed spot tests on some well-characterized archaea and did not find anything that would suggest anything but ‘random’ neighbors.

3. Some histone genes seem to belong to two categories. For example, in haloarchaea, in most cases, the acidic doublet "combination 3&4" and "single 3" type histones seem to map to the same gene (encoding the acidic doublet) – are there any cases where "single 3" is encoded in a gene by itself (i.e. not fused to 4)? Is this purposeful, or an error? Or do protein categories not correspond to encoding genes per se? Please correct or clarify how "strategies" (histone categories / clusters) are defined throughout. Answering this question would be easily addressed by adding a column of gene identifiers to the supplementary tables that correspond to the proteins (together with protein identifiers that exist in commonly used databases, see minor comments below) so that users could look them up.

If we understand this comment correctly, the reviewer assumes that combination 3&4 are fused in a single polypeptide chain, but they are in fact individual genes. So yes, there are cases where there is only a single cluster 3 histone, but the majority of the genomes appears to encode an additional cluster 4 histone. It is likely that the single 3 category might in fact be even smaller, as not all histones have been annotated. Each genome is listed only once, and has only one strategy.

We have added a column for the proteins which have a 100 % sequence match in UniProt to the excel table, but because the databases are largely unrelated, this could only be done for a few sequences since there is no good correspondence between uniprot and GTDB.

Minor comments:

1. For ease of comprehension, either the centroid histone or the best-studied histone from each cluster could be mentioned in the text, so that readers can put a "face" to a (cluster) name.

This is now included in figure 1b. We also added information to line 103 in the text.

2. "Closed tetramer" is undefined (line 253 and throughout).

Thank you for pointing this out. We added an explanation after the first occurrence (now line 260): *'Basic singlets from 'single 1' may form closed tetramers, as well as open, canonical tetramers. Closed tetramers are oligomeric histone assemblies in which one histone dimer contacts another dimer along the entire face of the dimer instead of just contacting through one 4HB (compare "Single 1" to "Single 2" in Supplemental Figure 8b). This is the case for our median histone sequence from the single 1 genome.'*

3. The results regarding Histones A and B from alphafold and MD appear to contradict (lines 264, 308). Can the authors expand on an explanation for this? Also, it would be better for clarity to explain what histone A and histone B are, before discussing these results.

We added an explanation to line 278: *Our representative from multiple 1 genomes encodes two basic histones, here referred to as "histone A" and "histone B". Only histone A is predicted to fold into the canonical tetramer. Histone B alone or combined with histone A, does not form a canonical tetramer in silico, but we did not explore whether this is a general feature of all histones in this cluster (Supplementary Figure 8c).*

4. Line 126: in the section "histones are used in 7 different strategies" – the phrase "acidic singlet (cluster 3)" is a typo, should probably read "acidic doublet".

Excellent catch! Fixed.

5. Please check the references throughout. There are some incorrect citations detected, especially in the intro, for example Line 69: Neither ref 32 nor 33 report ChIP-Seq data. Please reference Sakrikar et al., 2021 (https://doi.org/10.1093/nar/gkab1175) and 2023 (https://doi.org/10.1128/mbio.03449-22) for ChIP-seq with archaeal histones, and correct the text to say "…and ChIP-seq across haloarchaeal species…" (or add the other species that has been studied, i.e. Haloferax volcanii).

Thank you for pointing this out. We cited another paper by Amy Schmid's group there, but forgot to add the 2021 paper. Thank you for highlighting the 2023 reference. Text has also been amended.

6. To me, the language of "histone strategy" comes across as anthropomorphic while simultaneously being unclear whether the authors refer to a prediction or an experimentally tested mechanism. "Strategy" is used throughout together with verbs that describe actions of organisms, which should be clarified to ensure that the reader understands that everything is coming from predictions (e.g. introduction, "strategies' by which "organisms combine histones"; results line 113 section header "Histones are used…"; most of the figures…). I suggest that the authors use another descriptor to refer to the various combinations of histones encoded that is more aligned with what data the study is actually reporting. Example alternatives: "Seven different histone sets / groups / predicted DNA-protein complexes / types / categories are encoded in genomes"? There are many options that I think may more accurately refer to what they're reporting. Please amend the language throughout to avoid overstating claims.

While we appreciate that the term "Strategy" may come off as somewhat anthropomorphic, we think that it is an effective way to say that, for whatever reason, genomes have evolved to encode specific combinations of histones or only encode one, and that there is likely some underlying advantage to doing so. We also wanted to differentiate this terminology from the use of "Clusters" which we think would be more confusing. However, we have changed many instances in the text to be less anthropomorphic (highlighted in blue in the revised version). We do love our histones and might have been carried away a little bit.

7. Line 119 "one single" is redundant, should be "one" or "a single", please revise. Done

8. Protein and species identifier types seem to be inconsistent across ontologies in the supplementary data (RefSeq GCF ID's and GenBank GCA), different database protein identifier types (protein identifiers – what database are those from? Some are not detected by any searches on databases including GenBank, UniProt, EMBL, etc…). Related, how are the supplementary organism identifiers organized (all RefSeq IDs for all species and strains included, or just type strains / multispecies GenBank IDs that combines all strains of a species? If just type strains, why and how were these selected)? type species and / or species with experimentally characterized histones are missing in some cases. Some more careful checking and explanation of these supplementary files would be helpful to the community, and may lead to wider use of the dataset.

We BLASTed all proteins against UniProt databases and, wherever possible, added identifiers which were 100 % identical to the GTDB sequences to a new column in our spreadsheet. Many sequences have not made it to UniProt. We believe sourcing the GTDB is preferable because it allows for the exploration of additional predicted proteins not considered in Schwab et al., and because taxonomic information is annotated down to the species level. This allows us to extract information not available in the UniProt database.

Reviewer #3 (Remarks to the Author):

The manuscript "Histone diversity in the archaeal domain of life" by Laursen and Luger describes an in silico structural study of archaeal histones. First, all available archaeal genomes are searched for histone genes and 67% of known archaea contain histone genes. These histone genes were grouped in five clusters based on physical parameters and subsequently for each genome, the histone gene diversity was determined, and these were found across the whole archaeal phylogeny tree. Most genomes only contained one histone gene or multiple from the same cluster. Delving deeper into the structural features of these histone clusters using AlphaFold3, the predicted use of the four-helix bundle to form a tetramer was assessed as well as the presence of histone signature motifs in loop1 and loop2. Finally, the capability of these archaeal tetramers to form stable association with DNA was predicted and this was subsequently tested by MD simulations. As a final note, the authors state that "predictions are not yet ready to stand on their own without experimental validation." Overall, it is a clearly written manuscript with easy-to-follow figures. This study is of interest to the broader chromosome field by providing a structural = view of histone evolution in archaeal species. This reviewer only has a few concerns.

Major concerns:
+ The various archaeal histone genes were clustered based on physical parameters. Traditionally, genes are aligned and evolutionary relationship depicted in a phylogeny tree, and the accuracy of their relationship is tested by bootstrapping. If this latter approach is used, would this provide the same 5 groups of histones?

As suggested, we performed this analysis with our dataset from GTDB, **see new Supplementary Fig 11**. The clusters do not align well with those found with DBSCAN. This gives us confidence that our approach can pick up nuances that might be missed by only using CLANS. We think this provides an interesting alternative to solely sequence-based clustering.

+ On page 9, tetramer formation for multiple 1 and combination 1&2 are discussed, but it is unclear if four-helix bundle is formed in these cases based on the AlphaFold3 predictions. Please clarify this.

We removed the extra inset in Supplementary Fig 8 which might have caused the confusion. One of the histones from Multiple 1 forms the four-helix bundle, and two of the Combination 1&2 group form the four-helix bundle as well.

+ The cartoon for Supplemental Figure 6 is tricky to follow. A tetramer where loop 1 and loop 2 are highlighted would be more informative.

Thank you for the suggestion. We added "L1" and "L2" labels.

+ For the known structures in Supplemental Figure 8, were these also run in AlphaFold3 as controls? This is unclear from the figure and text.

The tetramers from the structures of the human and *M. fervidus* nucleosome are from the PDB and not predictions. Legend has been clarified "*a) Four-helix bundle structures from experimentally determined structures (not predicted through AlphaFold3).*" As is the case for other cluster 1 histones, alphafold predicts closed and open tetramer structures with similar ipTM scores.

Minor concerns: all done
+ Figure 1b, it would be informative if the percentage of the total genes is reported per cluster. – Done.
+ page 7, line 180 "… much divergent …" should be "… much more divergent …". – Done.
+ One page 7 line 209 to 8 line 211, a sentence in the middle of a paragraph is in bold. – Done.
+ The term "four-helix bundle" is abbreviated 4HB, but the term is only used seven times in total. Writing out four-helix bundle might be more accessible to non-histone aficionados. - Done.

Reviewer #4 (Remarks to the Author):

I co-reviewed this manuscript with one of the reviewers who provided the listed reports. This is part of the Nature Communications initiative to facilitate training in peer review and to provide appropriate recognition for Early Career Researchers who co-review manuscripts.
Thank you for your effort and for your thorough and fair review.

Reviewer #5 (Remarks to the Author):

The Authors provide in depth in silico analyses of the histone family in Archaea and the interactions of these proteins with DNA. Based on structure prediction, simulations and their in depth expertise in structures of the nucleosome(s), the survey yield five groups of histones based on their predicted structure and physical parameters. Groups 1 and 2 contain the typical histone fold typical of the well studied HMfB histone. Group 3 comprise two histone fold domain linked, Group 4 comprise short acidic proteins with truncation of helices 2 and 3 and group 5 contain four linked histone folds but lack many classic histone features and might have roles other than histones. There is no strong correlation of the presence of specific groups based on phylogeny, genome size, GC content and ecology, although as noted by the Authors, the paucity of informations on ecology and physiology limit this type of analysis. Importantly most archaeal histone are more acidic than their eukaryotic counterparts (exception group 1) and they show a universal conservation of residues in the L1 and L2 loops and groups 1 to 3 have the capacity to form homodimers but also heterodimers in genome encoding different types of histones 1 and 2 and even in the combination of group 3 and shorter histones of group 4. Various possible tetramers assembling two dimers on DNA are also evaluated with limitations of alphafold 3 overcome by molecular dynamics simulations indicating the likelihood of each combination.
Overall the study of of high interest for a broad and well written for a broad readership. There are only a series of minor comments that need to be addressed.

1. Fig2 illustrate that most genome contain a single type of histone. However the majority of genomes used in the study are not complete and originate from metagenomics and have not been manually curated. Hence it is likely that some genes including histone gene might be missing or that histones from groups 3 to 5 might have not been recognised as bona fide proteins and been removed from the genomes. So it would be important that the Authors remain caution about their conclusions. in addition, a very few species of archaea have been identified through cultivation while most remain "candidatus xxx". So the Authors should refer to genomes rather than species.

We indeed recognize that there could be mistakes in some of these sequences and that our findings are based on what is currently available. We changed two instances of "species" to genome. We have also added a statement / caution to this effect in several places in the manuscript.

2.Fig5b is crucial in the study and as pointed by the Author should be interpreted with perhaps more caution. Single 3 strategies look not so stable and it would be good that the Authors provide the simulations at 2KCl.

Thank you for this excellent suggestion. We repeated simulations for nucleosomes from predominately halophilic strategies (single 3, combo 3&4, and the cluster 3 histone only from combo 3&4) in 2 M KCl. We also repeated Multiple 1: histone A in 2 M KCl, as it had an average deviation above 20 Å from its starting structure, on par for the other halophilic nucleosomes. The results are shown in the **new Supplementary Figure 10**. It appears that the addition of 2 M KCl stabilizes the structures in silico. We have added new text at line 325.

3. line 340. As far as we understand, it is currently not possible to trace the similarity of archaeal histone to each type of eukaryotic histone. So it is logically not possible to directly compare the diversification from archaea versus eukaryotes in these terms. The Authors might rather state that as eukaryotes, archaea diversified their histones into subtypes.
We have reworded this statement (now line 360) to read: '*Intriguingly, 269 genomes encode an acidic and a basic histone that have < 60 % sequence identity, displaying a diversification in histone sequences similar to what might have happened during the diversification into H2A, H2B, H3 and H4 from a single histone precursor in early eukaryotes.*' We also included this reference:
https://doi.org/10.1093/bioinformatics/bty1000

4. As mentioned by the Authors, a broad survey of archaeal histone has been recently published based on in part similar criteria ref 14. It would be of high interest to the readers that the Authors compare the two studies in a Table showing overlap of the two classifications.

It is therefore not really possible to do a direct comparison between the two studies. However, we appreciate that the reader might be curious about this point. To address this, we attempted to outline major differences and similarities between the two studies in the new Supplementary Table 3. In the new Supplementary Table 4, we attempted to compare our five clusters to the clusters defined in Schwab et al., 2024 While two clusters likely represent the same types of proteins [Cluster 3 (Laursen) & Halo double (Schwab); and Cluster 5 (Laursen) & Poseidonia double (Schwab)], the other 3 clusters do not have direct analogs in the other study, but instead are likely dispersed among different clusters or not distinguished as different clusters in the other study.

The two studies used different datasets of proteins (InterPro vs GTDB) and there is only minimal overlap between the two (only about 200 proteins). It is therefore not really possible to do a direct comparison between the two studies. However, we appreciate that the reader might be curious about this point. To address this, we attempted to outline major differences and similarities between the two studies in the new Supplementary Table 3. In the new Supplementary Table 4, we attempted to compare our five clusters to the clusters defined in Schwab et al., 2024 While two clusters likely represent the same types of proteins [Cluster 3 (Laursen) & Halo double (Schwab); and Cluster 5 (Laursen) & Poseidonia double (Schwab)], the other 3 clusters do not have direct analogs in the other study, but instead are likely

dispersed among different clusters or not distinguished as different clusters in the other study. We discuss this starting line 412.

5.SupFig6 b the extension of the alpha2 helix might be erroneous because it overlaps with the RV motif normally in the L1.

We agree that this is difficult to see. We struggled to find a better angle, to no avail, and we therefore left the figure as is. The RKTV motif shares the R from the RD clamp, and although ChimeraX is showing a little turn there, the V (of I/L) doesn't normally extend into the next helix. As such, the decision made by chimeraX as to what to show as part of a helix is open to discussion.

Fig2i should be placed in position d and line 206 likely refers to the current pnal j, which should be also mentioned in line 203.

We assume the reviewer means supplementary figure 6. We placed the panels in this figure to first show the alignments of Single strategies first and then alignments from combinations, as is our convention throughout the paper.

You are correct about line 206, thanks!

6. line 317 typo on strength.- Done.

** See Nature Portfolio's author and referees' website at www.nature.com/authors for information about policies, services and author benefits.

This email has been sent through the Springer Nature Tracking System NY-610A-NPG&MTS

Confidentiality Statement:

This e-mail is confidential and subject to copyright. Any unauthorised use or disclosure of its contents is prohibited. If you have received this email in error please notify our Manuscript Tracking System Helpdesk team at http://platformsupport.nature.com .

Details of the confidentiality and pre-publicity policy may be found here http://www.nature.com/authors/policies/confidentiality.html

Privacy Policy | Update Profile

We thank the reviewers for an extensive review of our revised version. Below is our response to the last remaining comment from reviewer 1.

Reviewer #1 (Remarks to the Author):

We thank the author for the thorough consideration and response to our previous review. The previous concerns have mostly been addressed. One remaining concern still needs to be addressed (#5):

5. Reviewer comment: In silico DNA binding studies were done using the eukaryotic nucleosome length of 147bp. However, MNase data from various species usually suggests that archaeal histones typically bind DNA in ~60bp increments. The authors should try differing MNase digest lengths to make the simulations more physiologically relevant to what is already known about some archaeal histones. Author reply; We were explicitly looking for hypernucleosome structures, and therefore limited our simulations to 147 bp.

Can the authors clarify / justify in the main text why they used 147bp DNA in their AlphaFold structural predictions of histone-DNA interactions? The authors state in the introduction that 147bp is the length known to wrap eukaryotic nucleosomes, but their own structural work (Mattrioli et al., 2017) , in vivo MNase-seq of others (Ammar et al. 2012; Maryuama 2013) , in vitro studies from Reeve's group have all shown that the archaeal nucleosome wraps less DNA (for example, in Mattrioli et al, 90 bp for one nucleosome, 120bp for multiple stacked hypernucleosomes, sometimes less DNA in MNase-seq). I understand that the authors were explicitly looking for hypernucleosome structures and therefore I understand the need to use more than 60 bp in their structural precitions. But why did they use only one length of DNA with such a specifically eukaryotic bias (and not 60bp, 90bp, or other multiples thereof)? As previously requested, can the authors either provide a stronger justification for using 147bp and/or conduct the structural predictions with a more physiologically relevant DNA length?

We appreciate this reviewer's comment. The *in-silico* experiments described in this paragraph were directed at investigating whether closely packed hypernucleosomes are energetically favorable, or whether there was anything in the surface of any of the histones that would prevent this. Using 60 or 90 base pairs would not answer this question. To address this point, we added the following statement to the revised version of the manuscript.

We chose to run these predictions (and the simulations described below) with the canonical 147 base pair DNA fragment derived from Widom 601 for the following reasons: first, we wanted to provide enough length to probe the ability of archaeal histones to compact DNA in a closed hypernucleosome. Second, the Widom 601 sequence has a strong propensity to form nucleosomes *in vitro*. Third, because the sequence is the most prevalent in deposited nucleosome structures, models generated through AlphaFold3 using it as an input are biased towards nucleosome formation. As such, using this sequence would allow us to identify histones that are emphatically unable to form these compacted structures due to steric or electrostatic repulsion between two 'layers' of the hypernucleosome, and would allow for a direct comparison to eukaryotic nucleosomes.

Response to the remaining reviewer comment:

The text had already been updated in the previous version.

Text added (page 11):

We chose to run these predictions (and the simulations described below) with the canonical 147 base pair DNA fragment derived from Widom 601 for the following reasons: first, we wanted to provide enough length to probe the ability of archaeal histones to compact DNA in a closed hypernucleosome. Second, the Widom 601 sequence has a strong propensity to form nucleosomes *in vitro*. Third, because the sequence is the most prevalent in deposited nucleosome structures, models generated through AlphaFold3 using it as an input are biased towards nucleosome formation. As such, using this sequence would allow us to identify histones that are emphatically unable to form these compacted structures due to steric or electrostatic repulsion between two 'layers' of the hypernucleosome, and would allow for a direct comparison to eukaryotic nucleosomes.
